# Injectable cellular vesicle-based bone meal for inflammatory bone defect repair through restoring immune homeostasis

**DOI:** 10.7150/thno.110795

**Published:** 2025-03-18

**Authors:** Yu-Yue Zhao, Qian-Fang Meng, Hao Cui, Wei Liu, Yan Chen, Zhou Lan, Hao Chen, Dan-Dan Ma, Lang Rao, Guang-Tao Yu

**Affiliations:** 1 Stomatological Hospital, School of Stomatology, Southern Medical University, Guangzhou, 510280, China.; 2 Institute of Chemical Biology, Shenzhen Bay Laboratory, Shenzhen, 518132, China.

**Keywords:** MDSCs, Cell membrane vesicle, bone meal, bone repair, immune homeostasis

## Abstract

**Rationale:** Immune homeostasis microenvironment of bone regeneration is especially important for inflammatory-derived bone defect repair. The two key influencing factors for achieving ideal bone regeneration are the balance between inflammatory cells represented by T cells and anti-inflammatory cells represented by MDSCs, and the dynamic balance between osteoblasts and osteoclasts.

**Methods:** Herein, an injectable thermosensitive bone meal was designed with Pluronic F127 hydrogel loading myeloid-derived suppressive cells (MDSCs) membrane vesicles coated nano-hydroxyapatite (F127/nHA/MDSCs-MV, abbreviated as F127/nHAM) for periodontitis-derived bone defect repair.

**Results:** The proteomics revealed F127/nHAM were able to catalyze the production of adenosine from ATP depend on CD73 and CD39. *In vitro* and *in vivo* assays further showed that F127/nHAM inhibited the proliferation and function of T cells by component MDSCs-MV, exerting an anti-inflammatory role. Subsequently, the RNA-sequencing and confirmation experiments revealed that F127/nHAM inhibiting the differentiation of macrophages into osteoclasts through down-regulating the secretion of CCL2 and CCL5. In the periodontal bone defect rat model, the results of micro-CT and histological staining demonstrated that F127/nHAM had an outstanding anti-inflammatory and bone regeneration promoting properties, restoring immune homeostasis.

**Conclusion:** This biomimetic and multifunctional bone meal opens new avenues for inflammatory-derived bone defect repair and future clinical application.

## Introduction

Perturbation in local immune microenvironment can exacerbate inflammatory self-damage characteristic of many immune-mediated diseases 1. T cells is a pivotal driver in plenty chronic inflammatory diseases 2, 3. Myeloid-derived suppressive cells (MDSCs) are a group of negative immune regulatory cell populations that act as hostile forces against T cells and play a certain protective role in chronic inflammatory conditions 4-6. The imbalance between T cells and MDSCs further promotes the progression of the chronic inflammatory disease, such as periodontitis which act as one of the most common human diseases in which this mechanism is crucial 7. Dental plaque, as a driving factor of periodontitis, causes chronic inflammation, which leading to the destruction of periodontal tissue, absorption of alveolar bone, and even tooth loosening 8. In clinical and preclinical studies, the increase of T cells and decrease of MDSCs in gingival tissue from periodontitis lesion have been reported and this is associated with changes in up-regulation number of osteoclasts along with destruction of alveolar bone 9-12. Whereas T cells drive initial proinflammatory responses to environmental stimuli, MDSCs exhibit anti-inflammatory effects to promote inflammation remission and induce bone regeneration 13. Thus, modulating the balance of cell populations has an exciting potential to influence the etiology and fate of periodontitis.

Bone resorption, as one of the important criteria for diagnosing periodontitis, is mainly attributed to the excessive activity of osteoclasts exceeding the activity of osteoblasts 14. Osteoclasts originate from the hematopoietic mononuclear macrophage system and are a special type of terminally differentiated cell that can fuse its mononuclear precursor cells through various means to form large multinucleated cells 15. The OPG/RANKL/RANK signaling pathway has been confirmed to be the ultimate link in activating osteoclasts by numerous factors and plays an important role in alveolar bone metabolism 16. At present, the clinical treatment of bone defects caused by periodontitis mainly involves filling with commercial bone powder. In the later stage, through bone remodeling, new bone tissue is ultimately formed. After being well maintained, new bone tissue is ultimately formed through bone remodeling, which involves osteoclasts absorbing bone powder and osteoblasts forming new bone 17. Hydroxyapatite (HA), with extraordinary biocompatibility, appropriate biodegradability and osteo-inductivity, plays the primary and important component in commercial bone powder currently used in clinical practice 18, 19. Whereas, in the face of environmental stimuli in the oral cavity, commercial bone powder often leads to poor repair of alveolar bone, which caused by excessive activation of osteoclasts due to the lack of immune microenvironment regulation features in bone powder 20. Therefore, building an idealized bone powder that can regulate and restore the immune homeostasis is crucial for bone regeneration treatment.

Recent advances in nanotechnology have offered many new pathways for bone regeneration have been used to push the limit of current therapeutics 21, 22. Among them, cell membrane biomimetic nanotechnology has received widespread attention due to its natural origin and inheritance of protein functions from the maternal cell membrane 23. Owing to their excellent prolonging circulation time, resisting immunogenicity, reducing the toxicity of nanomaterials, protecting drugs from degradation, and specific targeting, cell membrane biomimetic nanotechnology has been widely applied in theranostic of diseases, including periodontitis 23. Currently, several types of cell membrane vesicles have been reported for modifying HA to improve osteogenic effects. Such as, engineering MC3T3-E1 cells membrane vesicles coated HA to facilitate initial steps of mineralization in osteoblasts 24. However, the regulatory effect of HA decorated by cell membrane vesicles on the immune cell homeostasis of periodontitis lesion that has been reported so far is not ideal.

**Scheme 1:**
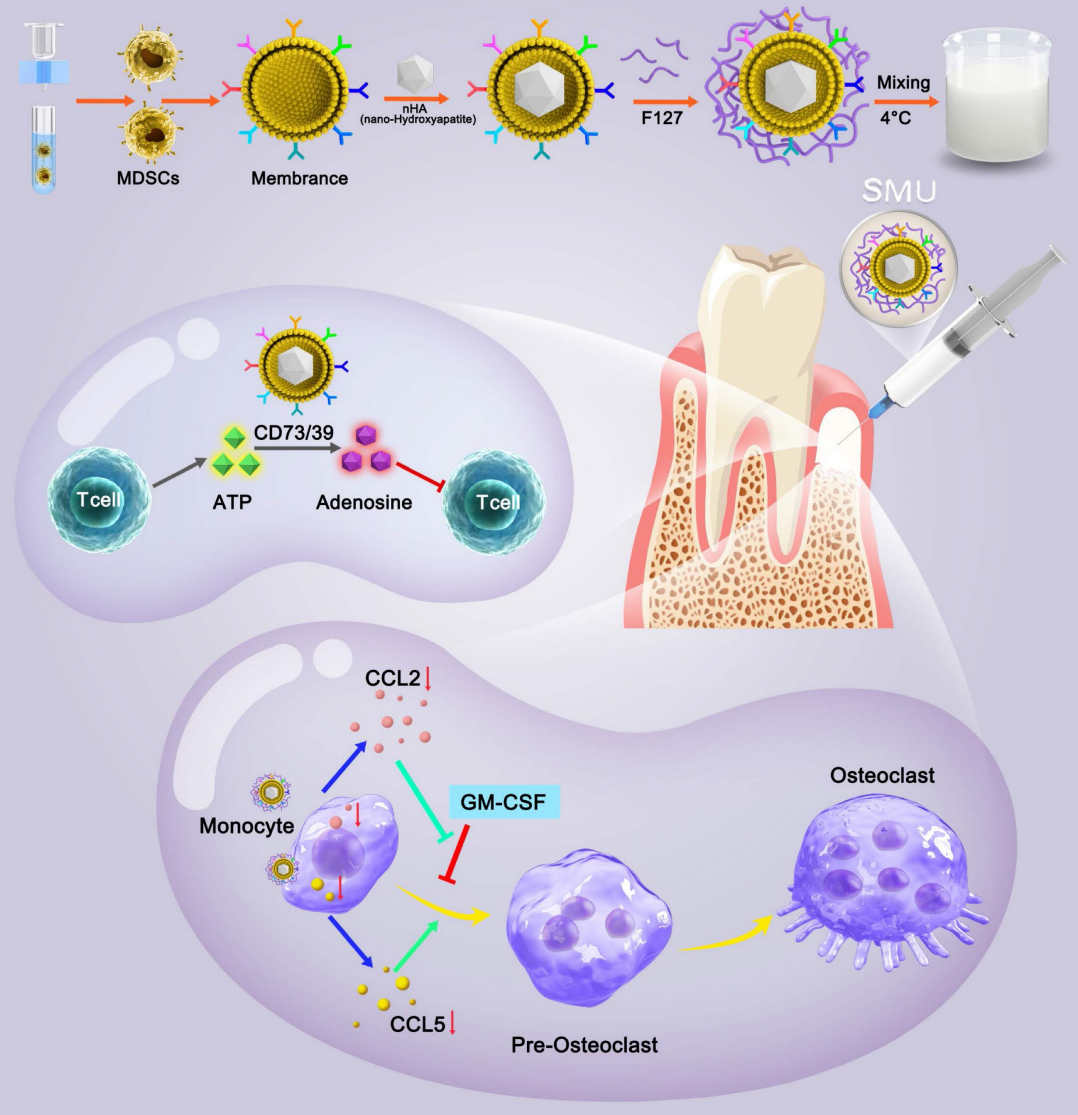
Schematic of preparation method and molecular mechanism of promoted bone regeneration of FA127/nHAM.

Pluronic F127 is an FDA approved non-ionic copolymer widely used for drug delivery 25. Given the unique characteristics of MDSCs for immune cells regulation, here we developed a novel thermosensitive injectable bone meal restoring immune homeostasis and promoting bone regeneration for periodontitis treatment. This system used MDSCs membrane vesicles as the outer shell and nano-HA (nHA) as the inner core to construct the vital component (nHA/MDSCs membrane vesicles, abbreviate as nHAM), and then loaded it into F127 hydrogel (the final product is named F127/nHAM) (Scheme 1). F127/nHAM had been confirmed to inhibit proliferation and functions of T cells via CD73/CD39-adenosina signaling pathway, which reversed inflammatory microenvironment and restored immune homeostasis. Meanwhile, F127/nHAM demonstrated the ability to down-regulation of osteoclasts by inhibiting the differentiation of mononuclear macrophages (osteoclast precursor cells). Furthermore, the RNA-sequencing revealed that the secretion inhibition of CCL2 and CCL5 caused by F127/nHAM inhibited the differentiation of mononuclear macrophages into osteoclasts (Scheme 1). As expected, by inflammatory scavenging and inhibited osteoclast differentiation, F127/nHAM significantly enhanced bone regeneration *in vivo*. The present study sheds light on a promising thermosensitive injectable bone meal for inflammatory bone repair.

## Materials and methods

### Preparation of NPs

The nHA power was purchased from Aladdin. The nHAM NPs were prepared by 0.2 mg nHA and 100 µL MDSCs membrane in 2 mL deionized water, and then magnetic stirring for 12 hours. Under 4 ℃ condition, 0.75 mL prepared F127 solution (27 wt%) was mixed with the 0.25 mL nHAM and stir magnetically for 12 hours to obtain F127@nHAM. Using the same method, 1 mL prepared F127 solution (20 wt%) was mixed with the 0.25 mg nHA to obtain F127/nHA. To construct F127/M, 0.9 mL prepared F127 solution (40 wt%) was mixed with the 100 µL MDSCs membrane and stir magnetically for 12 hours.

### Proteomics analysis of nHAM

To study the function of MDSCs membrane, three independent membrane samples were analyzed using proteomics. The MDSCs membrane was dissolved in TEAB buffer and sonication for 15 min. After centrifugation at 12000 r/min for 20 min, the supernatant was collected and added to a cold acetone containing 10 mM DTT. The mixture was then centrifugated at 12,000 r/min for 20 min to obtain the precipitate. In order to reduce the disulfide bonds, precipitate was mixed with 800 μL cold acetone and heated to 56 °C. The residue was collected by centrifugating at low temperature with 12,000 r/min for 20 min. Further Proteins digestion, desalting, iTRAQ labeling, fractionation proteomics LC-MS/MS analysis. To determine the biological and functional properties of all identified proteins, the identified protein sequences were analyzed on the basis of GO and KEGG terms.

### Evaluation of anti-inflammatory effect of F127/M in murine models of periodontitis

The murine models of periodontitis were constructed as above describe. After ligature removement, the F127/M (20 µL per mouse) was injected into lesion location every other day. At the endpoint, the gingival tissue from around of M1 were collected for further histological and immunological assays. Meanwhile, alveolar bone resorption level of the treated side of maxillae were detected by micro-CT.

### Treatment with F127/nHAM in rat models of periodontitis

For rat models of periodontitis, 8 - 10 weeks female SD rat were treated with ligature placement around subgingival area of M1 for 15 days. The silk sutures were checked once a week and was renewed in case of loosening or displacement. After the establishment of the periodontitis models, the ligatures were removed. And then, the 100 µL F127/nHAM was injected on days 0, 30 and 60 into lesion location. In the days 90, the alveolar bone resorption level of the treated side of maxillae were detected by micro-CT. Meanwhile, the periodontium specimens from around of M1 were collected for further histological assays.

### Statistical analysis

Graph Pad Prism version 9.0 for Windows (Graph Pad Software Inc, La Jolla, CA) was used for data analysis. Unpaired t test, one-way ANOVA with Dunnett's multiple comparison tests and two-way ANOVA with Tukey's or Sidak's multiple comparisons test was used to analyze significant differences. Dates were represented as the mean ± SEM. Differences (*p* < 0.05) were considered statistically significant.

## Results and Discussion

### MDSCs: alleviates the progression of periodontitis

**Figure 1:**
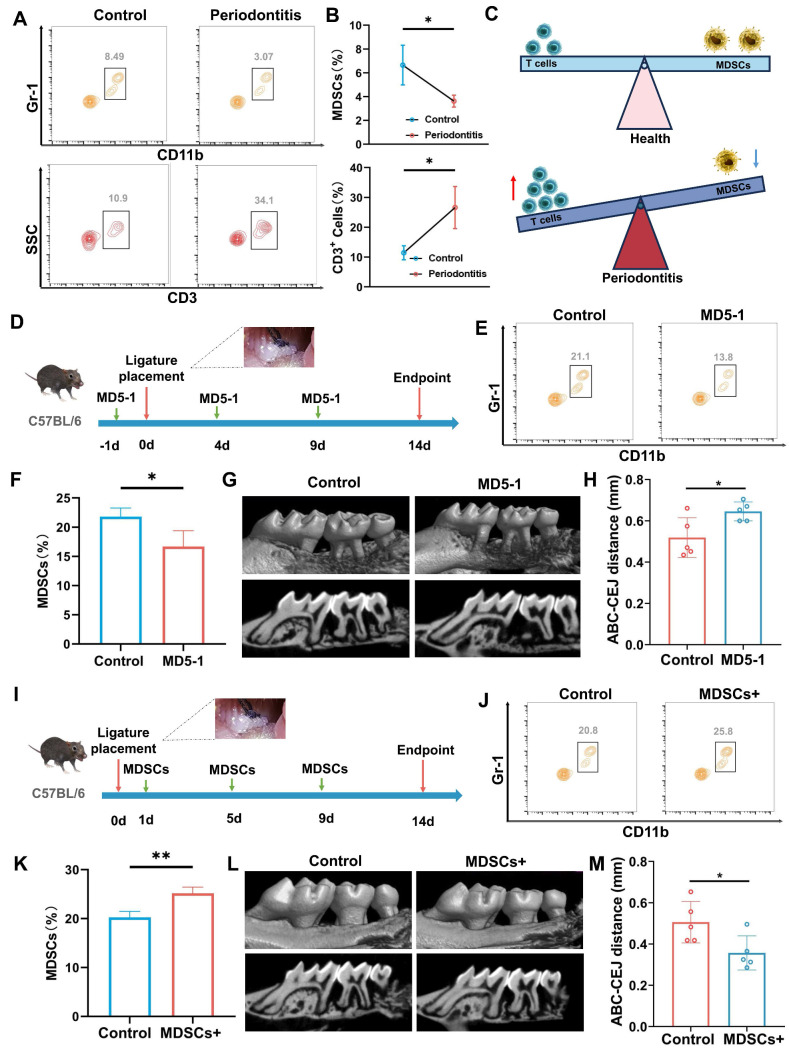
The regulatory role of MDSCs in the progression of periodontitis. A) Representative contour plot showing MDSCs and T cells in healthy or periodontitis periodontal tissue. B) The quantitative graph shows a significant increase in the number of MDSCs and the ratio of MDSCs to T cells in the periodontal tissue of mice with periodontitis. C) Schematic illustrating of MDSCs and T cells changes in physiology and periodontitis. D) Schematic illustrating of periodontitis progression animal assay under MDSCs elimination using MD5-1. E) Representative contour plot showing MDSCs in periodontal tissue of mice after PBS or MD5-1 treatment. F) The bar graph shows a significant decrease of MDSCs in blood of mice after MD5-1 treatment (*, p < 0.05). G). Representative micro-CT graphs show the aggravation of alveolar bone destruction after MD5-1 treatment. H) The quantitative graph shows a significant increase in ABC-CEJ distance in MD5-1 treatment group (*, p < 0.05). I) Schematic illustrating of periodontitis progression animal assay under MDSCs supplement. J) Representative contour plot showing MDSCs in periodontal tissue of mice after PBS or MDSCs supplement treatment. K) The bar graph shows a significant increase of MDSCs in blood of mice after MDSCs supplement treatment (**, p < 0.01). L) Representative micro-CT graphs showing the remission of alveolar bone destruction after MDSCs supplement treatment. H) The quantitative graph shows a significant reduce in ABC-CEJ distance in MDSCs supplement group (*, p < 0.05).

The occurrence of periodontitis is accompanied by the infiltration of inflammatory cells, which further promotes the progression of periodontitis 26, 27. In this study, we focused on the changes and roles of T cells and MDSCs in the progression of periodontitis. The number of T cells and MDSCs maintains a dynamic equilibrium in healthy periodontal tissue. When suffering from periodontitis, large numbers of inflammatory T cells migrate into periodontal tissues under the action of bacteria as the main driving factor. Meanwhile, the number of MDSCs that inhibit T cells was significantly downregulated (Figure 1A-C). The results of flow cytometry suggested that the reduction of MDSC weakens the inhibitory effect of T cells and promotes the progression of periodontitis. To further illustrate the role of MDSC in the progression of periodontitis, we conducted validation and rescue experiments. Targeting depletion of MDSCs using MD5-1 antibody validated its alleviating effect on periodontitis in the initial stage of modeling periodontitis (Figure 1D). The results of flow cytometry were used to confirm the effectiveness of MD5-1 in eliminating MDSC (Figure 1E-F). 3D-reconstructed micro-computer tomography (micro-CT) images and quantitative morphometric analyses were obtained at 2 weeks to assess bone destruction (Figure 1G-H). The results demonstrated that the reduction of MDSCs aggravated the progression of periodontitis. Next, the MDSCs were sorted from mouse bone marrow and injected into mice with periodontitis through the tail vein for rescue array (Figure 1I). Similarity Similarly, flow cytometry results confirmed an increase in MDSCs (Figure 1J-K). And, the bone destruction was also assessed by micro-CT) images and quantitative morphometric analyses (Figure 1L-M), which showed MDSCs alleviates the progression of periodontitis. To further explained that MD5 - 1 accelerates the progression of periodontitis by reducing the number of MDSCs, a replenishment experiment in which MDSCs are given after MD5-1 had been implemented (Figure S1A). The flow cytometry results demonstrated that the number of MDSCs remained relatively stable in MDSCs are given after MD5-1 treatment group (Figure S1B). Of course, there is no difference in bone destruction between MDSCs are given after MD5-1 treatment group and control (Figure S1C).

### Preparation and characterization of F127/nHAM

**Figure 2:**
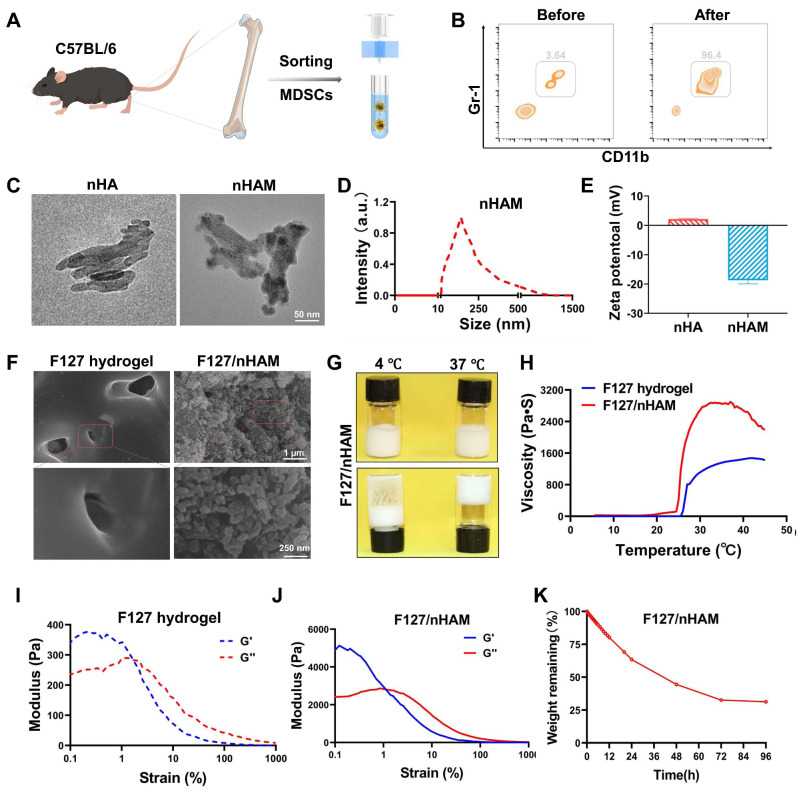
Preparation and characterization of F127@nHA. A) Schematic representation of sorting MDSCs from bone marrow using immunomagnetic beads. B) Representative contour plot showing MDSCs of before and after sorting. C) TEM images of nHA and nHAM (scale bar = 50 nm). D) Hydrodynamic diameter of nHAM by dynamic light scattering. E) Zeta potentials of nHA and nHAM. F) SEM of F127 hydrogel and F127/nHAM (scale bar =1 µm and 250 nm). G) Image of the flow state of F127/nHAM at 4 ℃ and 37 ℃ showing temperature sensitive characteristic. H) The SMU letters using F127/nHAM and the viscosity curve of F127 hydrogel and F127/nHAM. I) A strain-sweep rheological analysis of F127 hydrogel. J) A strain-sweep rheological analysis of F127/nHAM. K) The degradation curve of F127/nHAM.

Cell-membrane-coated nanoparticles have emerged as a promising therapeutic platform 23. The advancement of cell membrane-coating technique in inheriting the membrane protein profile of source cells inspired us to develop a MDSCs-mimicking bone meal to address the current challenges. To synthesize this bone meal, the MDSCs was firstly obtained from marrow of mouse and sorted using immunomagnetic beads (Figure 2A). Flow cytometry further checked the purity of MDSCs to ensure the synthetic quality of the nanomaterial (Figure 2B). Next, cells membrane was coated a nHA core with an average diameter of 110 nm to form the semi-finished nanoparticle nHA-MDSCs (nHAM) (Figure S2). The completeness of membrane coating was confirmed using transmission electron microscopy and showed a core-shell structure (Figure 2C). Dynamic light scattering (DLS) data displayed that the average hydrodynamic diameter of the nHAM was 120 nm and increased by ~20 nm compared with that of the uncoated nHA cores (Figure 2D). The nHAM remained stable over two weeks in FBS and PBS (Figure S3). The surface zeta potential changes of nHAM from positive to negative before and after the coating, which further indicated the success of the coating (Figure 2E). Sodium dodecyl sulfate-polyacrylamide gel electrophoresis (SDS-PAGE) stripes further revealed that MDSC membrane transfer did not influence the membrane protein expression in the MNP@MDSC (Figure S4).

In order to further expand the application potential of bone meal, we used F127 as a carrier for nHAM to construct an injectable bone meal (F127/nHAM). The microstructures of F127 and F127/nHAM were observed by SEM. We can clearly see a large number of grape beaded structure in F127/nHAM, which could facilitate osteoblast proliferation, migration and nutrients or metabolite transportation (Figure 2F). The flowability experiments in different temperature environments showed that F127/nHAM has the same temperature sensitive characteristics as F127 (Figure 2G and S5). Meanwhile, F127/nHAM can smoothly pass through a 29 G syringe, demonstrating excellent injectability (Figure S6). The curve of viscosity and the letter image of SMU also demonstrated the outstanding injectability of F127@nHAM (Figure 2H). And the compressive strengths of F127/nHAM and F127 were further assessed (figures 2I-J). Further, the F127/nHAM was placed in an environment with a constant temperature of 37 ℃ to evaluate the degradation behavior. The results demonstrated that after 4 days, the degradation ratio of bioink is up to percent 75 (Figure 2K). Above results suggested that our designed injectable bone meal is a promising nanomaterial for bone regeneration.

### Biological function of membrane components in F127/nHAM

**Figure 3:**
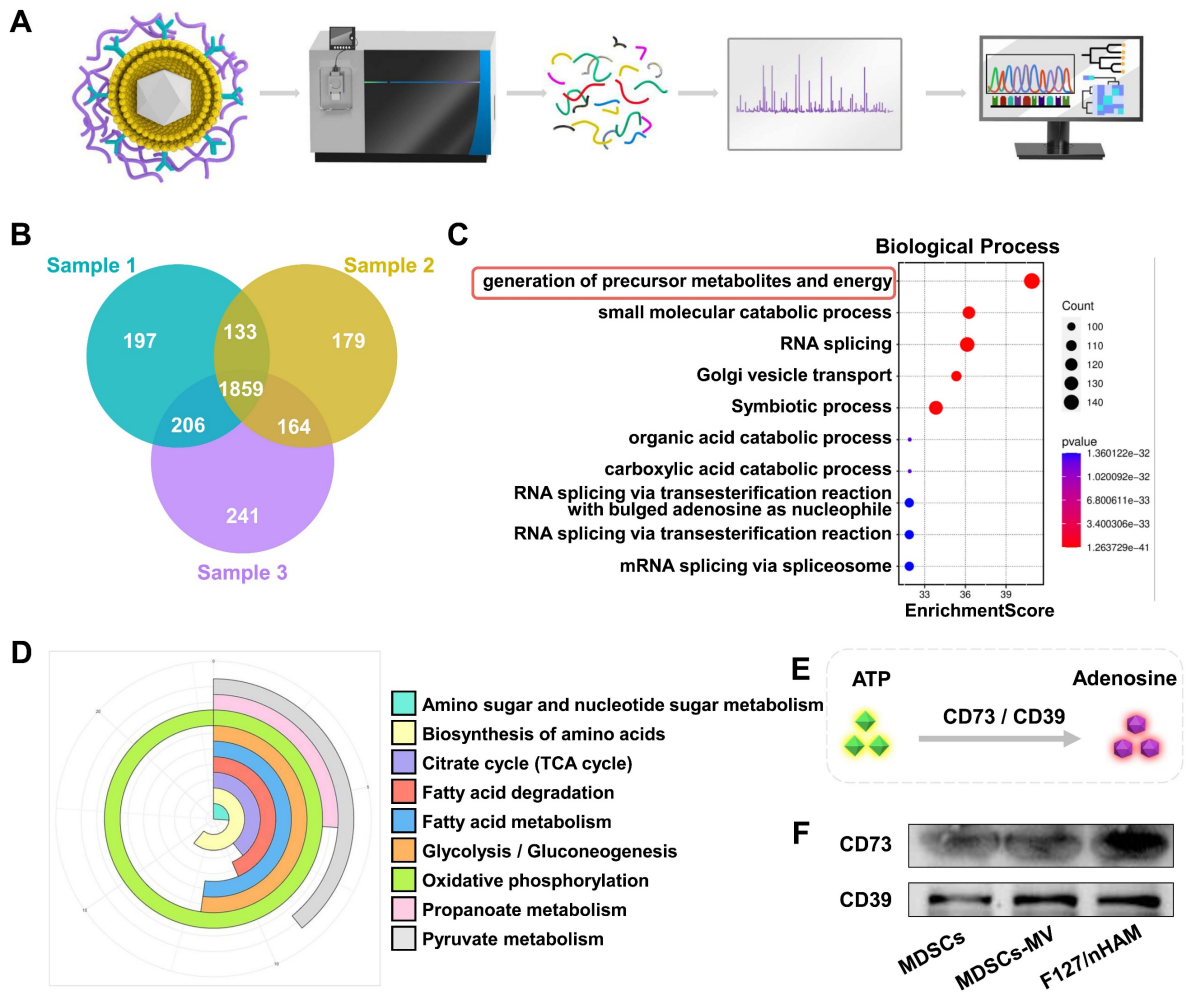
Proteomics analysis of membrane vesicle of MDSCs (MDSCs-CV). A) Schematic illustrating of proteomics analysis process. B) Venn diagram shows 1859 common protein components from three separate MDSCs-CV samples. C) The identified proteins were classified according to biological processes and analyzed through evolutionary relationships overrepresentation test with Fisher's exact test for significance. D) KEGG analysis of MDSCs-CV components. E) Schematic illustration of ATP metabolism generates adenosine via CD73 and CD39. F) WB confirms CD73 and CD39 exist on MDSCs, MDSCs-CV and nHAM.

The biological function of membrane components of obtained F127/nHAM were fist analyzed by proteomics as shown in Figure 3A. The Venn diagram of proteomics results showed that a total of 1859 protein components were detected simultaneously in three independent samples (Figure 3B). Gene Ontology (GO) biological process analysis indicated that the F127/nHAM components are involved in generation of precursor metabolites and energy processes (Figure 3C). The GO cellular component and molecular function analysis suggested that plentiful proteins and protein functions were closely related to metabolites and energy (Figure S7-8). Meanwhile, the Kyoto Encyclopedia of Genes and Genomes (KEGG) analysis also demonstrated that a series of signaling pathways were strongly linked with energy metabolism, such as oxidative phosphorylation, pyruvate metabolism and glycolysis / gluconeogenesis (Figure 3D). Based on results of proteomics and previous studies, we hypothesized that F127/nHAM were able to catalyze the production of adenosine from ATP depend on CD73 and CD39 (Figure 3E). In addition, western bolts results corroborated the presence of characteristic membrane proteins derived from MDSCs cell membranes, including CD73 and CD39, further indicating the potential for energy metabolism regulation from F127/nHAM (Figure 3F).

### F127/nHAM inhibits the proliferation and function of T cells

**Figure 4:**
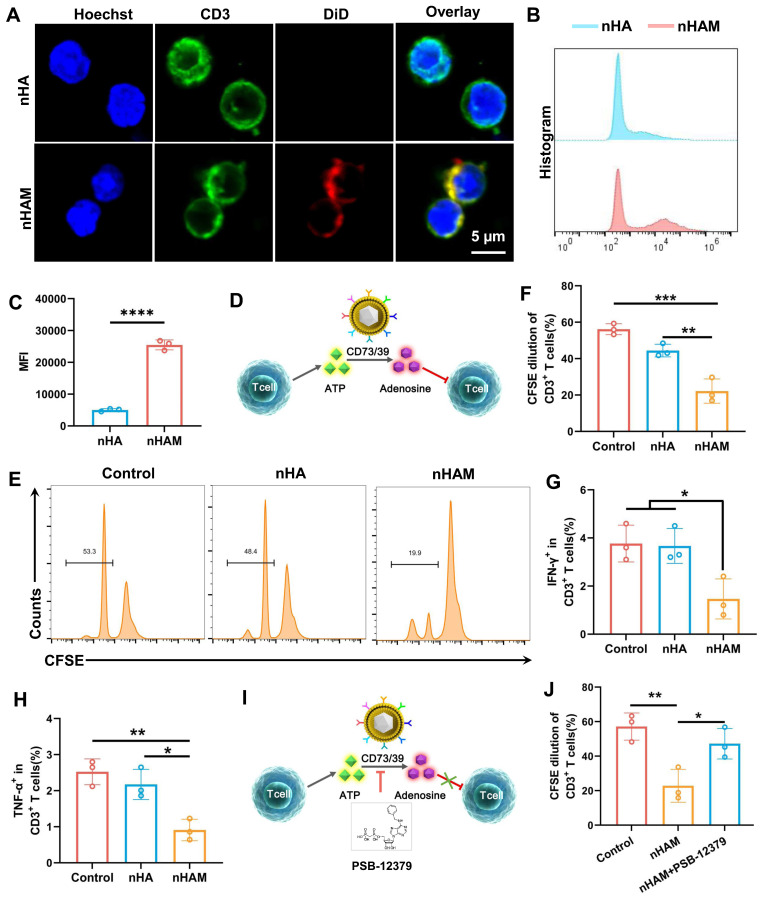
The inhibiting function of nHAM for T cells. A) Typical confocal images of CD3^+^ T cells incubated with nHA or nHAM (scale bar =5 µm). B) Representative flow cytometric plots of T cells bound with HA or nHAM. C) The quantitative graph shows nHAM specifically binds T cells (****, p < 0.0001). D) Schematic illustration of ATP metabolism generates adenosine via CD73 and CD39 from MDSCs-CV. E) Representative flow cytometric plots of the CFSE dilution of the labeled CD3^+^ T cells after incubation with nHA or nHAM with equivalent membrane proteins in the presence of CD3, CD28, and IL-2 for 96 h. F) The quantitative analysis of the CFSE dilution shows a significant inhibition for T cell proliferation in nHAM treatment group (*, p < 0.05). G-H) Fractions of IFN-γ^+^CD3^+^ T cells (G) and TNF-α^+^CD4^+^ (H) of total CD3^+^ T cells following cultivation with nHA or nHAM (*, p < 0.05; **, p < 0.01). I) Schematic illustration of the ATP metabolism generates adenosine promoted by MDSCs-CV is blocked via PSB-12379 (CD73 inhibitor). J) The quantitative analysis of the CFSE dilution shows the inhibition of nHAM on T cells proliferation was reversed by PSB-12379 (*, p < 0.05; **, p < 0.01).

To verify whether F127/nHAM can alleviate inflammation by inhibiting T cells in periodontitis, we directly conducted a co-incubation experiment with T cells and nHAM. Considering the carrier function of F127 and for the convenience of conducting experiments, we replaced F127/nHAM with nHAM *in vitro* assays. The splenocytes isolated from C57 mice were incubated with fluorescently labeled nHA and nHAM. The confocal imaging showed that there was a mass of nHAM tightly bound to the membrane of CD3^+^ T cells (Figure 4A), indicating specific interplay. Further, the splenocytes were also cultivated with the labeled nHA and nHAM to quantify the binding with CD3^+^ T cells. The results of flow cytometry displayed that nHAM group presented significantly higher MFI compare to nHA group, further verifying their close internation (Figure 4B-C). When nHAM acts on T cells, we deemed that it inhibits T cell proliferation and function by producing adenosine through CD73/CD39 mediated ATP metabolism as shown in Figure 4D. First, we evaluated the level of ATP and adenosine in T cells after nHAM treatment. The results demonstrated that the level of ATP and adenosine were significantly declined in nHAM treatment group compared to control (Figure S9A-B). Sequentially, we conducted carboxyfluorescein succinimidyl ester (CFSE) assay to evaluate the immunosuppressive effects of nHAM on effector T cells. The flow images and quantitative chart demonstrated that there were significantly lowered dilution ratios of CFSE-labeled CD3^+^ T cells in nHAM treatment group compared to nHA and control group, indicating that the proliferation of T cells was inhibited after nHAM treatment (Figure 4E-F). In order to better illustrate that nHAM can replace F127/nHAM in conducting experiments, we analyzed whether there are differences in the level of ATP, adenosine and proliferation CFSE between nHAM and F127/nHAM group. The quantitative analysis results confirm that replacing F127/nHAM with nHAM is very feasible (Figure S9). Flow cytometric analysis further displayed that nHAM prominently restricted tumor necrosis factor alpha-positive (TNF-α^+^) and interferon-gamma-positive (IFN-γ^+^) CD3^+^ T cells compared with those treated with nHA and PBS (Figures 4G-H), implying that nHAM could reduce cytokine production of T cells. Furtherly, the proliferation of T cells was evaluated by Ki67 staining. The results showed the same trend as CFST assay (Figure S10A). Additionally, the apoptosis of T cells is significantly downregulated after nHAM treatment (Figure S10B).

To verify that the inhibitory effect of nHAM on T cells depends on CD73/CD39 signal axis, we administered CD73 inhibitor (PSB-12379) and repeated the above CFSE assay (Figures 4I). Flow cytometric analysis showed that, different to nHAM alone group, nHAM + PSB-12379 group had almost no effect on proliferation of T cells ((Figures 4J). Therefore, we can conclude that F127/nHAM carrying CD39 and CD73 tended to closely aggregate on effector T cells to hydrolyze ATP released to pericellular sites, thereby potentially inhibiting the proliferation and inflammatory cytokine secretion of T cells.

While the MDSC membrane-nHA composite demonstrates originality in immunomodulated bone regeneration, we also evaluated the anti-inflammatory ability of nHA modified with macrophage and mesenchymal stem cell membrane (named Mac@nHA, MST@nHA separately). The results of CFSE staining declared that nHAM has stronger anti-inflammatory ability compared to Mac@nHA, MST@nHA, which also confirms the rationality of using MDSC membrane coating nHA in our project (Figure S11).

### F127/M alleviates the inflammation of periodontitis in mouse model

**Figure 5:**
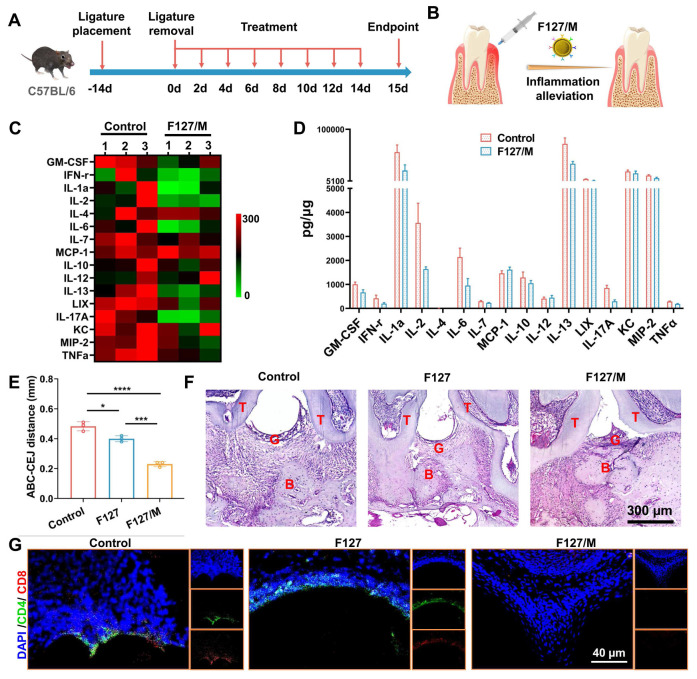
F127/M alleviates inflammatory responses in a periodontitis mouse model. A) Schematic illustration of model building and therapeutic design of F127/M in mouse. B) Schematic illustration of therapeutic effects of local F127/M administration on inhibiting inflammation. C) The heat map of Luminex liquid chip for cytokines. D) The quantitative analysis of the cytokines shows a significant alleviation for inflammatory response in F127/M treatment group, especially ILX, TNF-α, GM-CSF and MCP-1. E) The bar graph shows a significant reduce in ABC-CEJ distance in F127/M treatment group (*, p < 0.05; ***, p < 0.001; ****, p < 0.0001). F) Representative H&E-stained sections of periodontal tissue after different treatment (scale bar = 300 µm). G) Representative immunofluorescence-stained sections of periodontal tissue after different treatment (green: CD4; red: CD8; scale bar = 40 µm).

Considering the limitations of detecting the species of cytokine chip and in order to better describe the anti-inflammatory properties of MDSCs cell membrane, we prepared F127 hydrogel loaded with MDSCs cell membrane alone (F127/M). The temperature sensitivity experiment showed that F127/M had the same physical properties as F127/nHAM (Figure S12). Then, the efficacy of F127 to inhibit T cells to alleviate inflammation and periodontal tissue damage was evaluated in mouse models of periodontitis induced by ligature placement for two weeks. Subsequently, the F127/M were injected into the lesion location every other day to detect the possible alleviation of periodontal inflammation and tissue protection (Figure 5A-B). Mice treated with F127 or PBS was used as control. After continuous treatment for 15 days, the inflammation inhibition function of F127/M in the periodontal microenvironment was evaluated by Luminex liquid chip analysis of cytokines harvested from gum tissue. The heat map and histogram showed that almost all inflammatory cytokines from liquid chip panel were significantly inhibited after F127/M treatment in contrast to F127 group, including TNF-α and IFN-γ (Figure 5C-D). We once again verified the downregulation of TNF-α and IFN-γ after F127/M treatment by WB (Figure S13). These results suggested that F127/M had strong potential in alleviating inflammation in the gum of mice.

Furthermore, the molar area of maxillae was detected by microcomputed tomography (micro-CT) to assess the efficacy of F127/M in ameliorating bone resorption. Representative three-dimensional (3D) micro-CT views from the buccal side (Figure S14) and coronal two-dimensional (2D) view (Figure S15) showed severe alveolar bone loss around in the PBS group, suggesting the successful establishment of the advanced periodontitis model. Meanwhile, mice treated with F127/M had prominently higher alveolar bone levels compared to PBS groups. And, alveolar bone resorption in mouse treated with F127 was not alleviated compared to PBS group, indicating that anti-inflammatory and ameliorating bone resorption effect depends on MDSCs cell membrane, ameliorating bone resorption in F127/nHAM nanosystem. Measurements of the distance between the alveolar bone crest to cementoenamel junction (ABC-CEJ), an indicator of alveolar bone resorption, consistently verified that F127/M significantly reduced alveolar bone loss (Figure 5E). In addition, we conducted histological staining for maxillae. The results of hematoxylin and eosin (H&E) staining displayed that tissue sections from the PBS and F127 group showed more serious gum tissue destruction, disorder of periodontal tissue fibers as well as evident bone resorption compared to F127/M treatment group (Figure 5F and S16). Meanwhile, the representative immunofluorescence staining for CD4 and CD8 demonstrated that F127/M treatment significantly decreased the number of T cells in gum tissue microenvironment compared to both control group (Figure 5G and S17).

### nHAM inhibits differentiation in the direction of osteoclast

**Figure 6:**
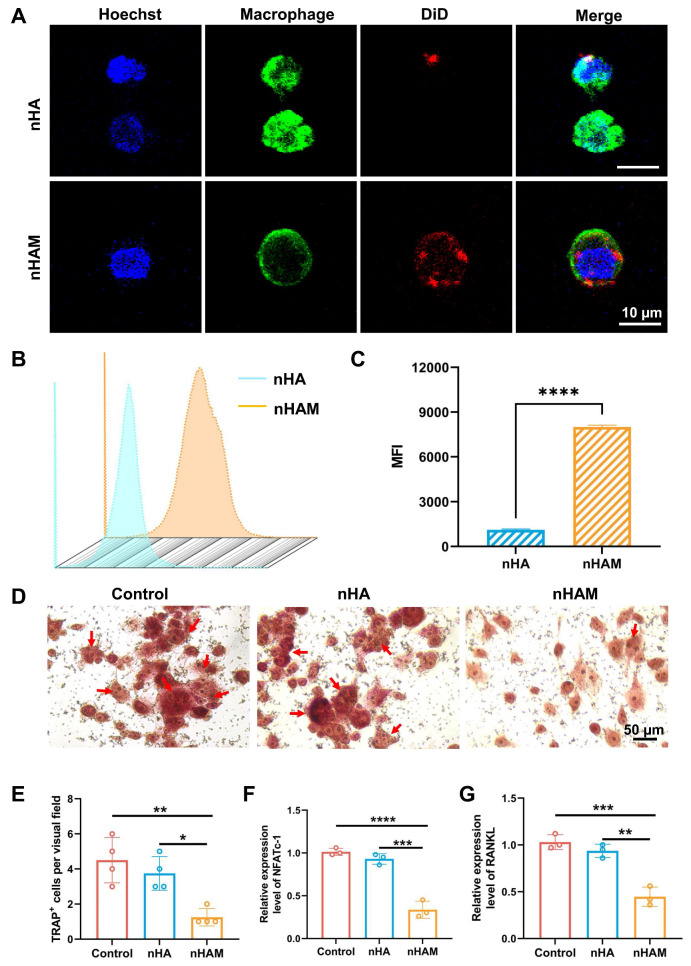
nHAM inhibits the differentiation of macrophages into osteoclasts in vitro. A) Representative confocal images of macrophages incubated with nHA or nHAM (scale bar=10 µm). B) Representative flow cytometric plots of macrophages bound with HA or nHAM. C) The quantitative graph shows nHAM specifically binds macrophages (****, p < 0.0001). D) Typical TRAP staining images of macrophages incubated with nHA, nHAM and blank control (scale bar = 50 µm). E) The quantitative bar graph about TRAP^+^ cell shows a significant inhibition for differentiation of macrophages into osteoclasts in nHAM treatment group (*, p < 0.05; **, p < 0.01). F-G) The bar plot of NFATc1 (F), RANKL1 (G) relative mRNA expressions in different treatment groups (**, p < 0.01; ***, p < 0.001; ****, p < 0.0001).

The dynamic changes of osteoclasts and osteoblasts play an important role in bone remodeling 28. Therefore, we want to investigate whether nHAM can inhibit osteoclasts to promote bone regeneration. Firstly, macrophages (Osteoclast precursor cells) were incubated with fluorescently labeled nHA and nHAM. The results of confocal imaging showed that more nHAM were bound to the surface of macrophages than nHA (Figure 6A), indicating nHAM directly bind to macrophages. Further, the nanoparticles binding with macrophages were also assessed by flow cytometry. The results displayed that nHAM group presented significantly higher MFI compared to nHA group, further verifying their close internation (Figure 6B-C). Subsequently, to observe the effect of nHAM and nHA on the differentiation of macrophages into osteoclasts through cell osteoclast induction experiments. Representative TRAP staining images and quantitative analysis of TRAP^+^ cells revealed that nHAM significantly inhibited the differentiation of macrophages into osteoclasts in contrast to nHA and blank control groups (Figure 6D-E). Further, gene expression analysis suggested that osteoclast-related genes, including NFATc-1and RANKL, were significantly downregulated in nHAM treatment group compared to nHA and blank control group (Figure 6F-G). The above data indicated that F127/nHAM has the potential to improve bone remodeling by inhibiting the differentiation of macrophages into osteoclasts.

### Biological mechanism of nHAM inhibiting the differentiation of macrophages into osteoclasts

**Figure 7:**
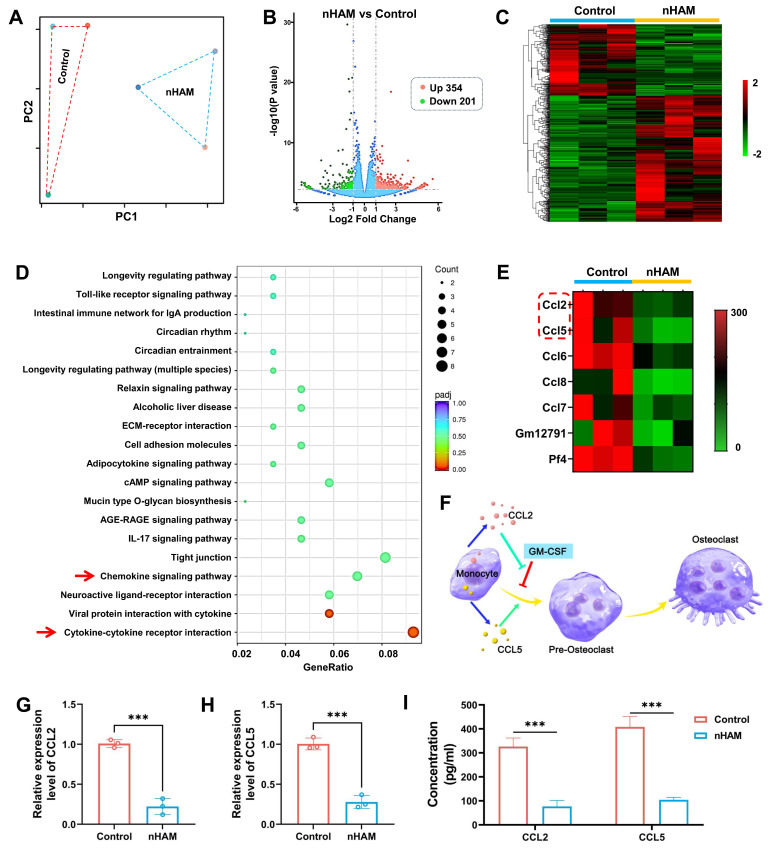
Transcriptomic analysis revealing nHAM negative regulation for differentiation of macrophages into osteoclasts. A) Spatial distribution of principal component analysis demonstrates homogeneity of each group of samples. B) Volcano plot of transcriptomic analysis of differentially expressed genes. C) Heatmap of differentially expressed genes which all up-regulated or down-regulated in nHAM and control groups (red: up-regulated; green: down-regulated). D) Down-regulated pathways in enriched KEGG terms of nHAM versus control analysis. E) The heatmap of down-regulated genes related to cytokines. F) Schematic illustration of osteoclast differentiation-associated cytokines (CCL2 and CCL5) in macrophages. G-H) The bar plot of showing a significant decrease of CCL2 and CCL5 mRNA expressions in nHAM treatment group (***, p < 0.001). I) The quantitative analysis of the secretion of cytokines CCL2 and CCL5 in macrophages incubated with nHAM and blank control (***, p < 0.001).

The RNA-sequencing was used to explore the underlying biological mechanism of nHAM for inhibiting the differentiation of macrophages into osteoclasts. Macrophages were treated with or without nHAM (three samples in each group). The PC distribution diagram and correlation analysis suggested high degree of uniformity among samples in each treatment group (Figure 7A and S18), ensuring the reliability of sequencing. Volcano plots and hierarchical cluster revealed that 555 genes are significantly different in the nHAM treatment group in contrast to control group, including 354 up-regulated genes and 201 down-regulated genes (Figure 7B-C). KEGG analysis indicated that Chemokine signaling pathway and Cytokine-cytokine receptor interaction (red arrow) was high enrichment (Figure 7D), indicating nHAM may affect the secretion of cytokines by macrophages. Coincidently, the GO analysis indicated that plentiful cytokines-associated biological process, cellular component and molecular function were significantly down-regulated after nHAM treatment (Figure S19).

Meanwhile, we further conducted statistical analysis on the expression of cytokine genes. The hierarchical cluster heatmap showed a series of genes were strongly reduced in nHAM group, including ccl2 and ccl5 (Figure 7E). Accumulated evidence suggests that CCL2 reverse and rescue osteoclast differentiation from GM-CSF repression and CCL5 strongly promotes osteoclast formation as shown in Figure 7F. So, we speculated that nHAM recovered osteoclast differentiation from GM-CSF repression by down-regulating CCL2 and reduced CCL5's promotion of osteoclast formation, thereby promoting bone regeneration. Further, the results of RT-PCR showed that the gene expression level of CCL2 and CCL5 were significantly decreased in nHAM treatment group in contrast to nHA and control group (Figure 7G-H). And, the ELISA assay consistently displayed significantly downregulated secretion of CCL2 and CCL5 after nHAM treatment (Figure 7I). Additionally, the situation of osteoclast formation after changing the expression levels of CCL2 or CCL5 has been detected by TRAP staining assay. The formation of osteoclasts was significantly promoted after CLL2 or CCL5 treatment, especially when used in combination, indicating that CCL2 and CCL5 promotes bone resorption (Figure S20).

### Anti-inflammation and osteogenesis mediated by F127/nHAM in rat model of periodontitis

**Figure 8:**
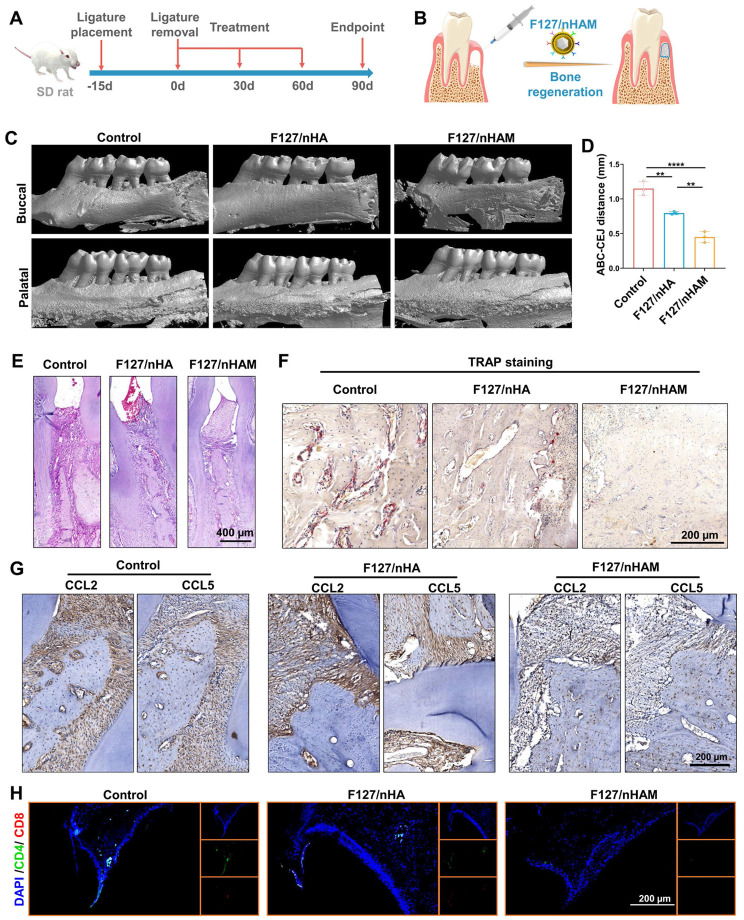
F127/nHAM promotes bone regeneration in a periodontitis SD rat model. A) Schematic illustration of model building and therapeutic design of F127/nHAM in SD rat. B) Schematic illustration of therapeutic effects of local F127/nHAM administration on improving bone regeneration. C) Representative micro-CT graphs of rat with F127/nHA, F127/nHAM or PBS treatment. D) The quantitative graph shows a significant decrease in ABC-CEJ distance in F127/nHAM treatment group (**, p < 0.01; ****, p < 0.0001). E) Representative H&E-stained sections of therapeutic area tissue after different treatment (scale bar = 400 µm). F) Representative TRAP-stained sections of therapeutic area tissue after different treatment. G) Representative immunofluorescence-stained sections of therapeutic area tissue after different treatment (green: CD4; red: CD8; scale bar = 200 µm). H) Representative immunohistochemistry-stained for CCL2 and CCL5 of therapeutic area tissue after different treatment (scale bar = 200 µm).

The toxicity of F127/nHAM was detected through co incubation experiments with HOK cells. The CCK results showed that F127 had little effect on epithelial cells (Figure S21). Next, a preclinical rat model of ligature-induced periodontitis was established to evaluate the effect of F127/nHAM on the mitigation of periodontal inflammation and improving bone remodeling. Ligature placement for two weeks was used to induce periodontitis and then temperature-sensitive bone meal of F127/nHAM or F127/nHA as well as PBS were injected at bone resorption site once every month after ligature removal, based on the degradation characteristics of the material and the osteogenic effect evaluation of our previous administration (Figure 8A and S22). After continuous treatment for three months, the therapeutic effect on anti-inflammation and bone regeneration of F127/nHAM was evaluated (Figure 8A-B). Representative 3D micro-CT views from both buccal (Figure 8C) and coronal 2D view (Figure S23) indicated that F127/nHAM and F127/nHA treatment both promoted bone regeneration to vary degree in contrast to control group, with F127/nHAM showing the best therapeutic effect. Measurements of ABC-CEJ consistently verified that F127/nHAM significantly promoted alveolar bone regeneration (Figure 8D), indicating that MDSCs cell membrane components enhance the efficiency of bone regeneration for nHA.

In addition, we conducted histological staining for maxillae. The results of hematoxylin and eosin (H&E) staining demonstrated that tissue section from the PBS group showed more serious gum tissue destruction, disorder of periodontal tissue fibers as well as evident bone resorption. Differently, tissue sections from F127/nHAM and F127/nHA displayed relatively intact gingival papilla, relatively neat periodontal tissue fibers and plenty of interalveolar bone, especially in F127/nHAM group (Figure 8E and S24). Representative TRAP staining images further confirmed that F127/nHAM effectively inhibited osteoclast activity to improve bone regeneration at alveolar bone around ligature sites (Figure 8F). The above sequencing results reveal that F127/M inhibits osteoclast formation by reducing the secretion of CCL2 and CCL5. Therefore, we detected the expression of CCL2 and CCL5 in each experimental group via immunohistochemistry staining. The results confirmed that the secretion of CCL2 and CCL5 were prominently reduced in F127/nHAM treatment group compared to F127/nHA and control group (Figure 8G). Besides, the representative immunofluorescence staining for CD4 and CD8 demonstrated that F127/nHAM treatment significantly decreased the number of T cells in gum tissue microenvironment compared to both control group (Figure 8H). Meanwhile, we also assessed the inflammation cytokines in periodontium tissue microenvironment. The representative staining images revealed that F127/nHAM significantly inhibited the secretion of IFN-γ and TNF-α in contrast to F127/nHA and control group (Figure S25 and S26). Finally, we evaluated blood biochemical indicators to detect whether the treatment had significant toxic side effects. The quantitative analysis results showed that the treatment of F127/nHAM and F127/nHA has no side effect in preclinical rat model (Figure S27). Taken together, our funding provided evidence that F127/nHAM alleviated inflammatory responses and promote alveolar bone regeneration, suggesting them as an alternative bone meal to treat periodontitis and highlighting their potential for further clinical translation.

In this project, we used rodent models to validate the effectiveness and explore the mechanism. Although the similarity between mouse and human genes is high, differences still exist 29. Further validation in non-human primates such as rhesus monkeys can better confirm the feasibility and effectiveness of our material's clinical translation. In addition, the large-scale preparation of MDSCs, one of the components of the F127/nHAM, poses certain difficulties, such as high costs. On the other hand, due to individual tissue compatibility issues, it is necessary to prepare membrane vesicles of MDSCs by oneself in future clinical applications, which belongs to the category of personalized customization and has high requirements for medical institutions, making it difficult to promote at the grassroots level. Although the F127/nHAM still has a long way to go before it can enter clinical applications, the future is bright.

## Conclusion

In summary, we have developed a novel thermosensitive injectable bone meal for inflammatory-derived bone defect treatment via restoring immune homeostasis and promoting bone regeneration. The F127/nHAM to have excellent properties in inhibiting proliferation and functions of T cells depending CD73/CD39-adenosina signaling pathway, which reversed inflammatory microenvironment and restored immune homeostasis. Furthermore, F127/nHAM showed the ability to down-regulation of osteoclasts by inhibiting the differentiation of mononuclear macrophages (osteoclast precursor cells). Meanwhile, the RNA-sequencing revealed that the secretion inhibition of CCL2 and CCL5 caused by F127/nHAM inhibited the differentiation of mononuclear macrophages into osteoclasts. Noteworthily, F127/nHAM shows a superior bone regeneration ability than nHA by inflammatory scavenging and inhibited osteoclast differentiation* in vivo*. Ours study sheds light on a promising thermosensitive injectable bone meal for inflammatory bone repair.

## Supplementary Material

Supplementary experimental section, figures.
