# Supplementary material for: Injectable cellular vesicle-based bone meal for inflammatory bone defect repair through restoring immune homeostasis
Source: Theranostics. 2025 Mar 18;15(10):4465–80. doi: 10.7150/thno.110795 (PMC11984383; doi:10.7150/thno.110795)
Supplement: Supplementary file 1 — Supplementary experimental section, figures. [file thnov15p4465s1.pdf]

Supporting Information

Injectable cellular vesicle-based bone meal for inflammatory bone defect repair through restoring immune homeostasis

Yu-Yue Zhao[#], Qian-Fang Meng[#], Hao Cui, Wei Liu, Yan Chen, Zhou Lan, Hao Chen, Dan-Dan Ma*, Lang Rao*, Guang-Tao Yu*

Experimental Section

Animals

Female six- to eight-week-old C57BL/6 mice and eight- to ten-week-old SD rat were purchased from Experimental Animal Center of Southern Medical University and bred under specific pathogen-free (SPF) conditions in the animal center at stomatology of hospital. All experimental procedures were approved and performed in accordance with the guidelines of stomatology of hospital of southern medical university.

Evaluation of local immune microenvironment in murine models of periodontitis

To construct murine models of periodontitis, 6-8 weeks old C57BL/6 mice were treated by silk sutures (8-0) placement into the round subgingival area of M1 for 14 days. The silk sutures were checked once a week and was renewed in case of loosening or displacement. After the establishment of the periodontitis models, the ligatures were removed, and the mice were used to study the numbers of MDSCs and T cells by flow cytometry.

For MDSCs deletion assay, the time of silk suture was set to day 0. The MD5-1 (MDSCs inhibitor) was injected on days -1, 4, 9 and 14 via intraperitoneal injection method. In the days 14, the number of MDSCs were evaluated to confirm the effectiveness of MD5-1. In addition, alveolar bone resorption level of the treated side of maxillae were detected by micro-CT.

For MDSCs intervening assay, firstly, the silk sutures were placed and the isolation of MDSCs were delivered to mice via the tail vein on days 1, 5 and 9. Similarly, the number of MDSCs were evaluated to confirm that the number had increased at the days 14. Meanwhile, alveolar bone resorption level of the treated side of maxillae were detected by micro-CT.

For replenishment experiment in which MDSCs are given after MD5-1, the time of silk suture was set to day 0. The MD5-1 was injected on days -1, 4, 9 and 14 via intraperitoneal injection method. And the isolation of MDSCs were delivered to mice via the tail vein on days 1, 5 and 9. Similarly, the number of MDSCs were evaluated to confirm that the number had increased at the days 14. Meanwhile, alveolar bone resorption level of the treated side of maxillae were detected by micro-CT.

Isolation of MDSCs

MDSCs Isolation Kit (mouse) was purchased from Miltenyi Biotec (German). Firstly, the femur was dissected from tumor-bearing C57BL/6 mouse. Then, the cells in bone marrow cavity were collected by mans of irrigation and then filtered with 70-μm cell strainers (Becton & Dickinson). Next, the cell

suspense was indirectly magnetically labeled with Anti-Gr-1-Biotin and anti-biotin microbeads. Then, the cell suspension is loaded on to a MACS Column, which is placed in the magnetic field of a MACS Separator. The magnetically labeled cells are retained with the column. After removing the column from the magnetic field, the magnetically labeled cells were collected in a 15 ml centrifuge tube. To increase the purity of MDSCs, repeat the magnetic separation procedures as above steps. Finally, cells were fluorescently stained with CD11b (BD, BB515, clone M1/70) and Gr-1 (BD, BV650, clone RB6-8C5) and analyzed by flow cytometry to confirm the purity of MDSCs. Cell debris and dead cells were excluded from the analysis based on scatter signals and propidium iodide fluorescence.

Cell membrane collection

Briefly, MDSCs was disrupted using a Dounce homogenizer. The entire solution was subjected to 20 passes before spinning down at 3200g for 5 min. The supernatants were saved and centrifuged at 20000g for 30 min, after which the pellet was discarded and the supernatant was centrifuged again at 80000g for 2 h using an ultra-speed centrifuge (LE-80K, Beckman Coulter, USA). The pellets containing the cell membranes were washed once in 1 mM EDTA and 10 mM Tris-HCl, and then collected as purified MDSCs membranes. Then MDSCs membrane-vesicles were obtained by physically extruding the pellets for several passes through 400 nm and 200 nm microporous membranes with an Avanti mini-extruder (Avanti Polar Lipids, USA). According to the BCA method, approximately 100 million MDSCs yielded 1 mg of membrane material (protein weight). The MDSCs membranes were suspended in PBS containing 0.2 mM EDTA at a protein concentration of 2 mg/mL and stored at -80 ℃ for further use.

Preparation of NPs

The nHA power was purchased from Aladdin. The nHAM NPs were prepared by 0.2 mg nHA and 100 µL MDSCs membrane in 2 mL deionized water, and then magnetic stirring for 12 hs. Under 4 ℃ condition, 0.75 mL prepared F127 solution (27 wt%) was mixed with the 0.25 mL nHAM and stir magnetically for 12 hs to obtain F127@nHAM. Using the same method, 1 mL prepared F127 solution (20 wt%) was mixed with the 0.25 mg nHA to obtain F127/nHA. To construct F127/M, 0.9 mL prepared F127 solution (40 wt%) was mixed with the 100 µL MDSCs membrane and stir magnetically for 12 hs.

Characterization of NPs

The morphological properties of the nHA NPs and the nHAM were observed using transmission electron microscopy (TEM, Talos F200X) operated at an acceleration voltage of 200 kV. The size

distribution, stability and zeta potential were evaluated using Zetasizer Nano-ZS (Malvern Instruments). For F127 and F127/nHAM morphological properties, the freeze-dried NPs were detected by scanning electron microscope at an accelerating voltage of 30 kV (SEM, JSM-IT300). The viscosity and modulus of F127 and F127/nHAM were assessed using rheometer (ARES-G2) within the temperature range of 0-50 ℃ and the strain range of 0.1-1000, respectively.

Proteomics analysis of nHAM

To study the function of MDSCs membrane, three independent membrane samples were analyzed using proteomics. The MDSCs membrane was dissolved in TEAB buffer and sonication for 15 min. After centrifugation at 12000 r/min for 20 min, the supernatant was collected and added to a cold acetone containing 10 mM DTT. The mixture was then centrifugated at 12,000 r/min for 20 min to obtain the precipitate. In order to reduce the disulfide bonds, precipitate was mixed with 800 μL cold acetone and heated to 56 °C. The residue was collected by centrifugating at low temperature with 12,000 r/min for 20 min. Further Proteins digestion, desalting, iTRAQ labeling, fractionation proteomics LC-MS/MS analysis. To determine the biological and functional properties of all identified proteins, the identified protein sequences were analyzed on the basis of GO and KEGG terms.

Western blotting

Western blotting was performed according to our previously study.[1] In brief, 1×SDS-PAGE loading buffer was used to lyse the membrane samples and then the lysates were resolved by SDS-PAGE and transferred to polyvinylidene difluoride (PVDF) membranes. Afterward, the PVDF membranes were incubated with CD73, CD39, TNF-a and IFN-γ (CST). The protein level was examined using chemiluminescence detection reagents (Merck Millipore, Billerica, MA).

Cell culture

RAW 264.7 mouse macrophages were obtained from the American Type Culture Collection (ATCC, Manassas, VA) and genotype confirmed using STR sequence. The cells were cultured in Dulbecco's modified Eagle's medium (DMEM), 10% fetal bovine serum (FBS), at 5% CO2 and 37 ° C in a humidified incubator according to ATCC guidelines.

Adhesion assay of nHAM for T cells

Splenocyte suspension were obtained from C57BL/6 mice. The poly-L-lysine (1 mg/mL, Sigma-Aldrich) was used to wash dishes twice at room temperature. And then the splenocyte cells were seeded onto dishes at a dose of 5×10^6 per well and incubated with 0.1 mL of 0.2 mg/mL DiD-labeled nHA

and nHAM at 4 °C for 1 h. Afterward, the dishes were washed with PBS three times and fixed with 4% paraformaldehyde (PFA) at 4 °C for 0.5 h. Subsequently, 3% BSA were added into dished to block nonspecific binding sites for 0.5 h. Then, FITC-labeled CD3 (BioLegend) and Hoechst 33342 were used to stain T cells. After PBS washes, the slides were observed using confocal microscopy (Leica TSC SP8). In order to further analysis of specifical adhesion capacity of nHAM to T cells, nHAM and nHA were similarly co-cultured with splenocyte cells in 24-well plates at 4 °C for 1 h. Then, these cells were collected, washed with PBS and stained with FITC-CD3 antibody (BioLegend). These cells were washed again and analyzed using flow cytometry.

T cells proliferation, activation and apoptosis assay

Splenocyte cells were obtained from C57BL/6 mice and seeded into 96-well plates in a density of 5×10^6 per mL. And then, CFSE (5 μM, Invitrogen) dyeing reagent was added to mark T cells. Afterward, anti-CD3 (1.5 mg/mL), anti-CD28 (1 μg/mL) and IL-2 (200 U/mL) were added into plates to activate naïve T cells for successive 4 days. Meanwhile, nHA and nHAM were added into plates in a density of 0.2 mg/mL and replenished every other day. After 5 days culture, these cells were collected, washed and stained with BV510-CD3, as well as intracellular stained with APC-IFN-γ, PE-TNF-a after membrane rupture, which analyzed the proliferation activity and secretion of inflammatory factor of T cells. For further explanation of CD73/CD39 signaling dependence, simultaneously perform PSB-12379 (CD73 inhibitor) treatment during nHAM treatment and repeat the above experiment and detection. For apoptosis assay, the cells were stained with annexin V-FITC and propidium iodide solution (both from BD Biosciences) for 15 min at RT in the dark and immediately analyzed by flow cytometry.

Evaluation of anti-inflammatory effect of F127/M in murine models of periodontitis

The murine models of periodontitis were constructed as above describe. After ligature removement, the F127/M (20 μL per mouse) was injected into lesion location every other day. At the endpoint, the gingival tissue from around of M1 were collected for further histological and immunological assays. Meanwhile, alveolar bone resorption level of the treated side of maxillae were detected by micro-CT.

Cytokine microarray

Mouse High Sensitivity T Cell Magnetic Bead Panel (#MHSTCMAG-70K, Merck Millipore) was used and all experimental operations are carried out according to the manufacturer's instructions. In brief, 96-wells plates were coated with 200 μL wash buffer and shocked at room temperature for 10 min. 50 μL of standard or quality control substance was added to the corresponding well; 50 μL MXMSM-11 was added to the background well; 25 μL of Assay Buffer was added to the sample well; 25 μL of the

sample to be tested was added to the corresponding sample well; and then 25μL of microbeads was added to all wells. Afterward, the 96-wells plates oscillated on the oscillator at 2-8 ℃ away from light overnight. 25 μL of detection antibody and 25 μL SAPE sequentially added to all well. And then, plates were washed with buffer three times. At last, 150 μL of Sheath Fluid or Drive Fluid were added to all wells for further analysis via Luminex®200TM.

Adhesion assay of nHAM for macrophages

The poly-L-lysine (1 mg/mL, Sigma-Aldrich) was used to wash dishes twice at room temperature. And then RAW264.7 was seeded onto dishes at a density of 5 $\times 10^6$ per well and incubated with 0.1 mL of 0.2 mg/mL DiD-labeled nHA and nHAM at 4 ° C for 1 h. Afterward, the dishes were washed with PBS three times and fixed with 4% paraformaldehyde (PFA) at 4 ° C for 0.5 h. Subsequently, 3% BSA were added into dished to block nonspecific binding sites for 0.5 h. Then, FITC-labeled CD11b (BioLegend) and Hoechst 33342 were used to stain macrophages. After PBS washes, the slides were observed using confocal microscopy (Leica TSC SP8). In order to further analysis of specifical adhesion capacity of nHAM to macrophages, nHAM and nHA were similarly co-cultured with Raw264.7 in 24-well plates at 4 °C for 1 h. Then, these cells were collected, washed with PBS and analyzed using flow cytometry.

Macrophage-osteoclast differentiation assay

Macrophages were obtained from bone marrow cells according to previous study. And then, macrophages were seeded into 96-wells plates in a density of 1 $\times 10^4$ per well in culture medium supplemented with MCSF (30 ng/mL) and RANKL (50 ng/mL). Meanwhile, these cells were simultaneously treated with 0.2 mg/mL nHA and nHAM. The group without treatment acted as control. Seven days after, the evaluation of osteoclast differentiation was checked using TRAP staining kit (Kamiya, China), in which multinucleated TRAP$^+$ (more than three, purple color) cells were confirmed as osteoclasts. Additionally, macrophages were seeded into 24-wells plates and treated with 100 μL of 0.2 mg/mL nHA and nHAM in the culture environment of MCSF (30 ng/mL) and RANKL (50 ng/mL) for three days. Afterward, the cells were collected to evaluate the relative mRNA expression of NFATc-1 and RANKL via RT-PCR. In order to furher identify the role of CCL2 and CCL5, macrophages were seeded into 24-wells plates and treated with CCL2 or/and CCL5.

RNA-sequencing

To further explore the biological mechanism of macrophage-osteoclast differentiation, Macrophages are induced to undergo MCSF (30 ng/mL) and RANKL (50 ng/mL) treatment and given 0.2 mg/mL nHAM simultaneously for three days. The RNA-sequencing operation was carried out according to our

previous study.[2] In brief, the total RNA was extracted and quantified. Sequencing libraries were generated using NEBNext® UltraTM RNA Library Prep Kit for Illumina® (NEB, USA) following manufacturer’s recommendations and index codes were added to attribute sequences to each sample. Gene Ontology (GO) enrichment analysis of differentially expressed genes was implemented by the cluster Profiler R package. GO terms with corrected P value less than 0.05 were considered significantly enriched by differential expressed genes. KEGG is a database resource for understanding high-level functions and utilities of the biological system from molecular-level information. Cluster Profiler R package was used to test the statistical enrichment of differential expression genes in KEGG pathways.

Treatment with F127/nHAM in rat models of periodontitis

For rat models of periodontitis, 8- 10 weeks female SD rat were treated with ligature placement around subgingival area of M1 for 15 days. The silk sutures were checked once a week and was renewed in case of loosening or displacement. After the establishment of the periodontitis models, the ligatures were removed. And then, the 100 μL F127/nHAM was injected on days 0, 30 and 60 into lesion location. In the days 90, the alveolar bone resorption level of the treated side of maxillae were detected by micro-CT. Meanwhile, the periodontium specimens from around of M1 were collected for further histological assays.

Flow cytometry

For flow cytometric detection, single cell suspensions were firstly prepared from cells with different treatment. Subsequently, the cells were stained with following antibodies: Live/Dead (AF700), CD45 (APC-Cy7; clone 30-F11), CD3 (BV510, clone 145-2c11), CD11b (APC; clone M1/70), Gr-1 (BV650; clone RB6-8C5), IFN-γ (PE; clone GIR-208), TNF-a (FITC; clone MP6-XT22), Ki67 (PE; clone B56) (all from Becton & Dickinson). The specimens were measured by a CytoFLEX flow cytometer (Beckman). The results were analyzed using FlowJo (Tree Star).

Micro-CT analysis

To assess the level of alveolar bone resorption, the resected mouse and rat maxillae were fixed in 4% paraformaldehyde for 24 hs and then soaked in 75% ethyl alcohol for following micro-CT scanning (Scango).

Histological staining assays

After micro-CT analysis, the mouse and rat tissue specimens were decalcified in 10% EDTA for 20 and 45 days, respectively. And then, the tissue specimens were dehydrated and embedded in paraffin. Then

serial sections were cut along the mesio-distal direction. Afterward, the sections were used for H&E, TRAP and immunofluorescence staining. The primary antibodies for our experiment included the following: CD4 (CST, 92599) and CD8 (CST, 98941).

ELISA

Macrophages were seeded into 96-wells plates in culture medium supplemented with MCSF (30 ng/mL) and RANKL (50 ng/mL). Meanwhile, these cells were simultaneously treated with 0.2 mg/mL nHAM. After 7 days, the OD values of the corresponding liquids were measured at 450 nm with an ELISA kit in an enzyme marker (Novatein Biosciences) for CCL2 and CCL5. For metabolic process of T cells, ATP and adenosina were alos detected. All operations are performed according to the manufacturer’s instructions.

RT-PCR

Quantitative real-time PCR was performed in triplicate with an Applied LightCycler 96 quantitative PCR system (Roche). The primer and probe sequences were as supplemental **Table 1**.

Statistical analysis

Graph Pad Prism version 9.0 for Windows (Graph Pad Software Inc, La Jolla, CA) was used for data analysis. Unpaired t test, one-way ANOVA with Dunnett’s multiple comparison tests and two-way ANOVA with Tukey’s or Sidak’s multiple comparisons test was used to analyze significant differences. Dates were represented as the mean ± SEM. Differences (p < 0.05) were considered statistically significant.

References

1. Lan Z, Zou KL, Cui H, Zhao YY, Yu GT. Porphyromonas gingivalis suppresses oral squamous cell carcinoma progression by inhibiting MUC1 expression and remodeling the tumor microenvironment. Mol Oncol. 2024; 18: 1174-88.
2. Cui H, Zhao YY, Wu Q, You Y, Lan Z, Zou KL, et al. Microwave-responsive gadolinium metal-organic frameworks nanosystem for MRI-guided cancer thermotherapy and synergistic immunotherapy. Bioact Mater. 2024; 33: 532-44.

Supporting Table and Figures

Table S1. Primer used in RT-PCR

Gene	Forward primer (5’ to 3’)	Reverse primer (5’ to 3’)
β-actin	GTGACGTTGACATCCGTAAAGA	GCCGGACTCATCGTACTCC
NFATc-1	GAGAATCGAGATCACCTCCTAC	TTGCAGCTAGGAAGTACGTCTT
RANKL	GGAAGCGTACCTACAGACTATC	AAAGTGGAATTCAGAATTGCCC
CCL2	TTTTTGTCACCAAGCTCAAGAG	TTCTGATCTCATTTGGTTCCGA
CCL5	CTCGCTGTCATCCTCATTGCTA	GCACTTGCCACTGGTGTAGAAA

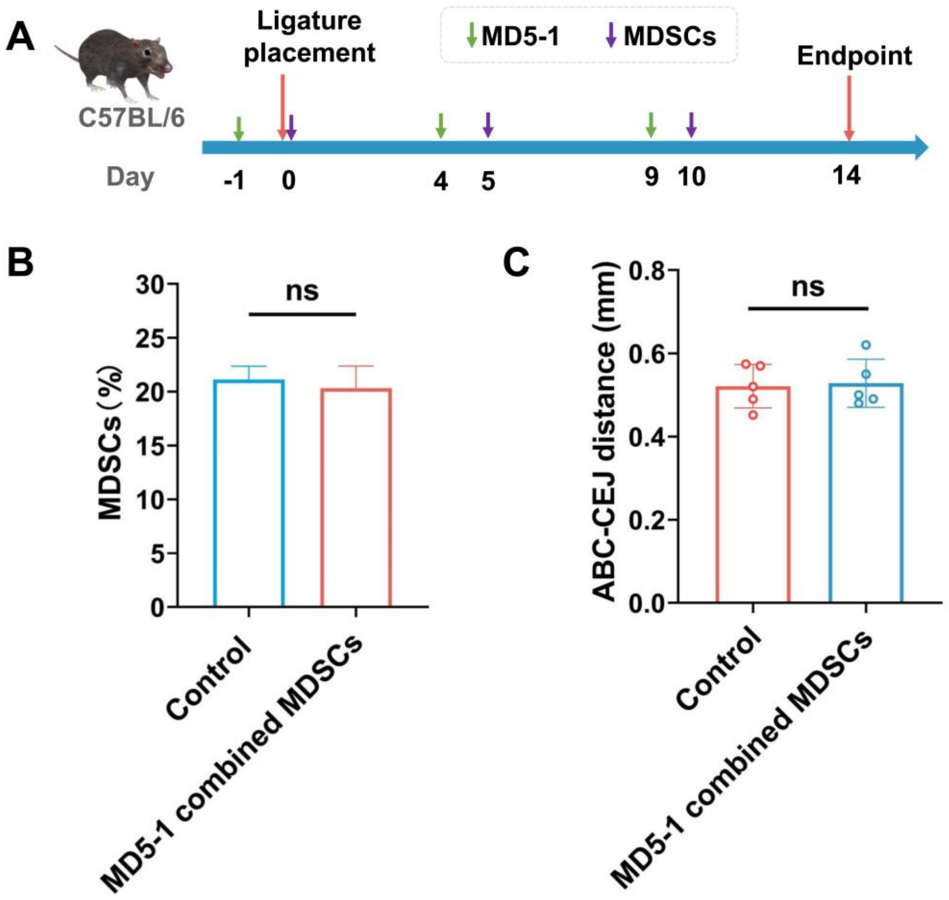

Figure S1. A) Schematic illustrating of periodontitis progression animal assay under MDSCs elimination using MD5-1 combined with MDSCs supplement. B) The bar graph shows the changes of MDSCs in blood of mice in different treatment groups. C) The quantitative graph shows ABC-CEJ distance in the treatment group was basically consistent with that in the control group.

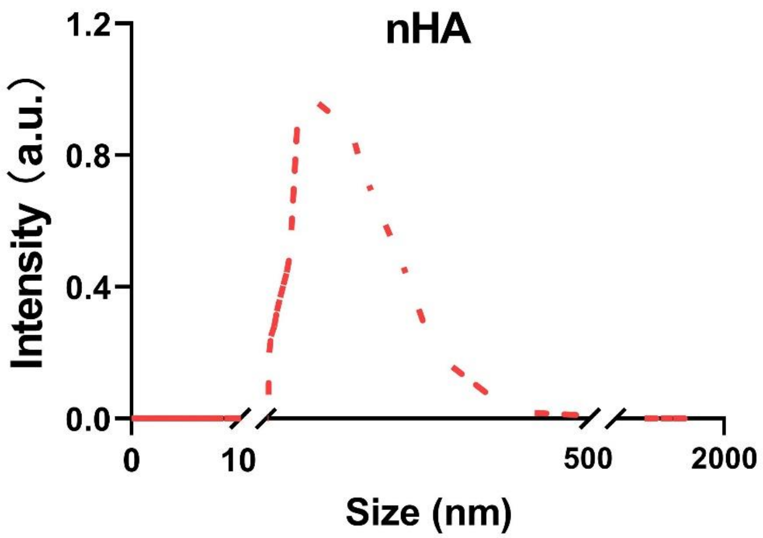

Figure S2. Hydrodynamic diameter of nHA by dynamic light scattering.

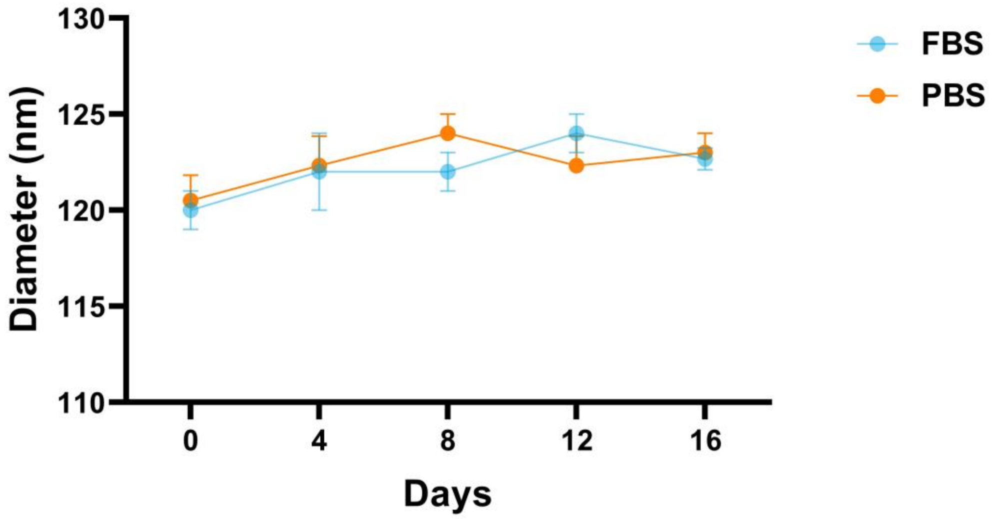

Figure S3. The stability of nHAM: hydrodynamic diameter of nHAM dispersed in FBS or PBS for 14 d measured by dynamic light scattering.

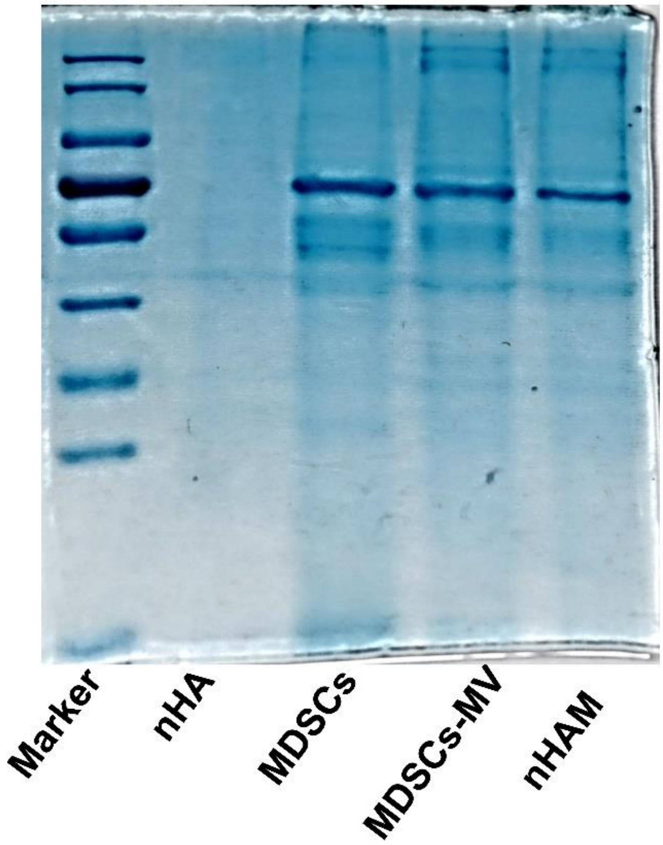

Figure S4. Coomassie Blue Staining of nHA, MDSCs, MDSCs-CV and nHAM.

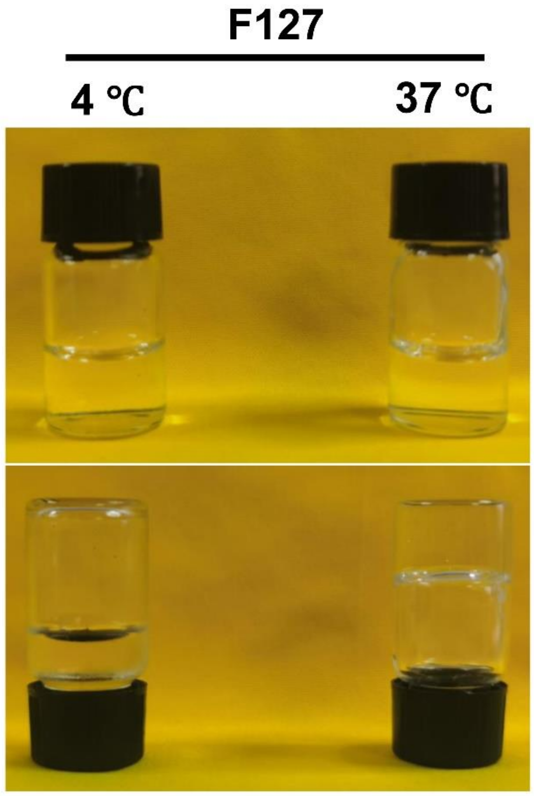

Figure S5. Image of the flow state of F127 at 4 ℃ and 37 ℃ showing temperature sensitive characteristic.

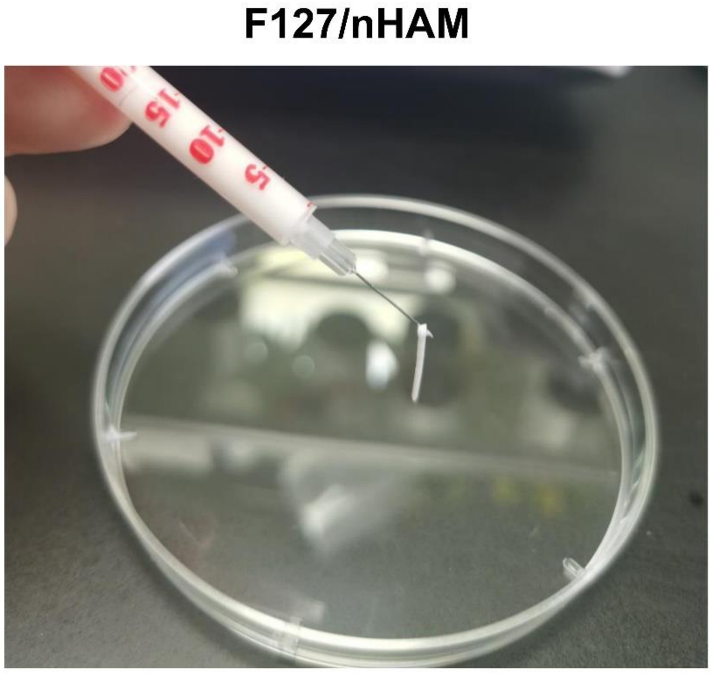

Figure S6. Injectability of F127/n HAM.

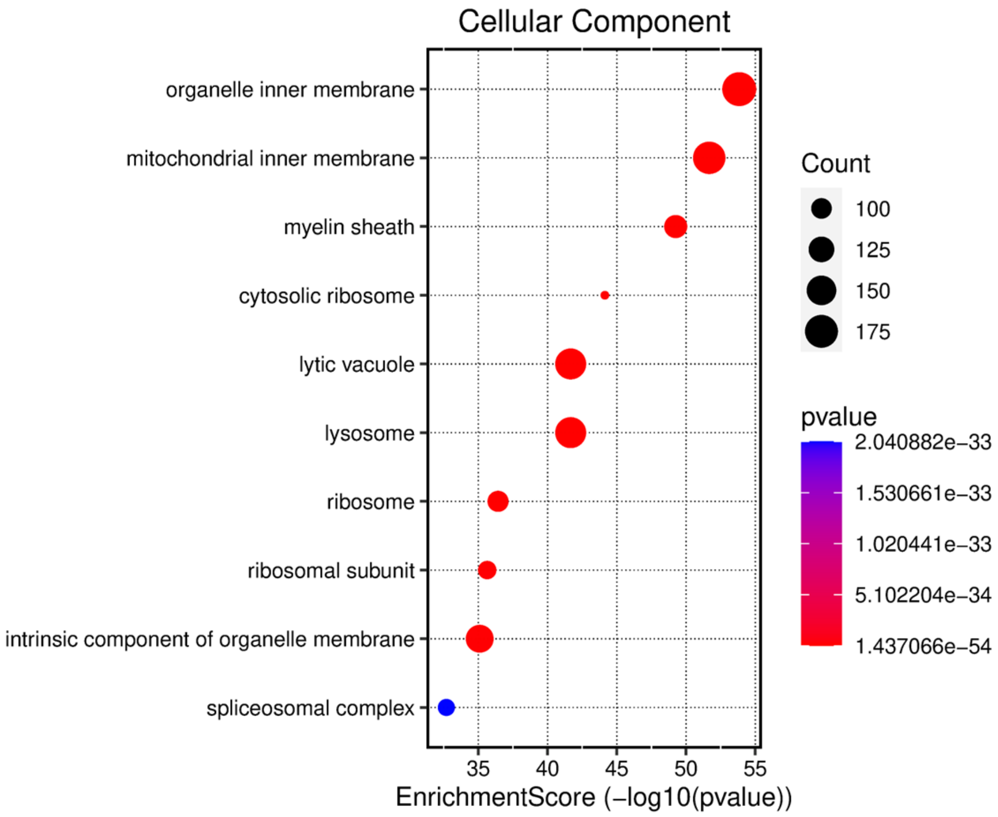

Figure S7. Cellular component analysis of proteomics of MDSCs-CV.

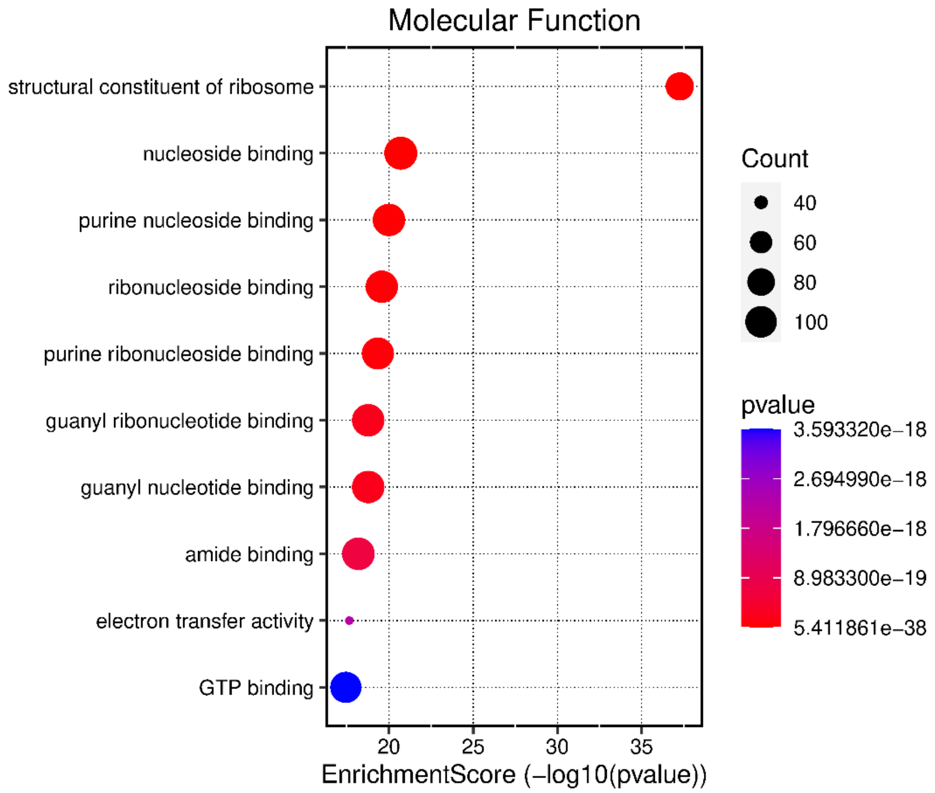

Figure S8. Molecular function analysis of proteomics of MDSCs-CV.

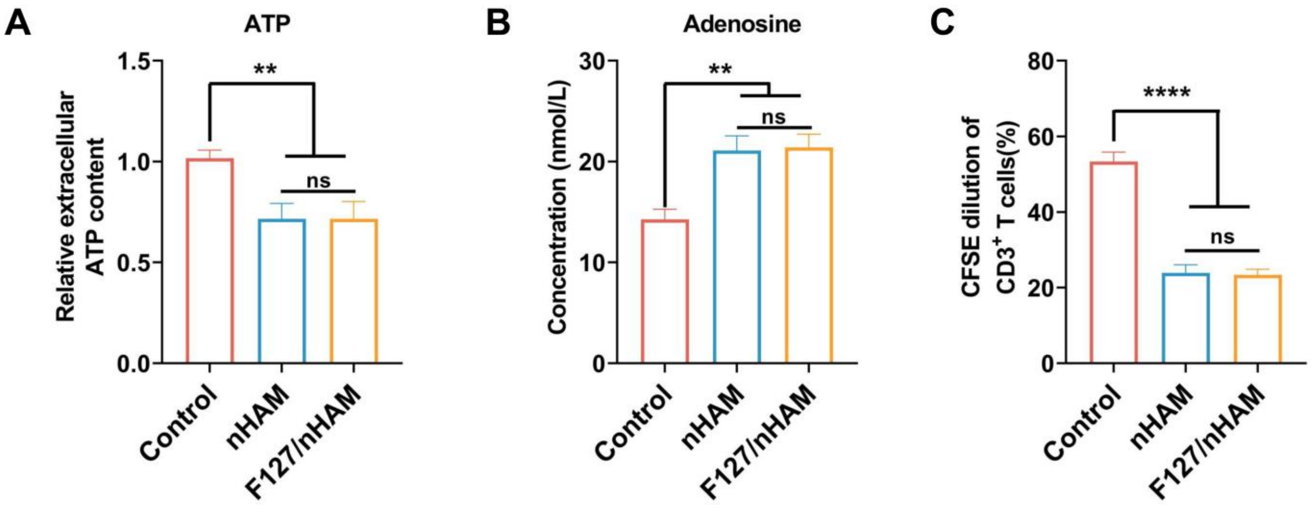

Figure S9. A) The quantitative analysis of relative extracellular ATP content in T cells after incubation with nHAM, F127/nHAM and blank control (**, p < 0.01). B) The quantitative analysis of adenosine content in T cells cultivation system after incubation with nHAM, F127/nHAM and blank control (**, p < 0.01). C) The quantitative analysis of the CFSE dilution in T cells cultivation system after incubation with nHAM, F127/nHAM and blank control (****, p < 0.0001).

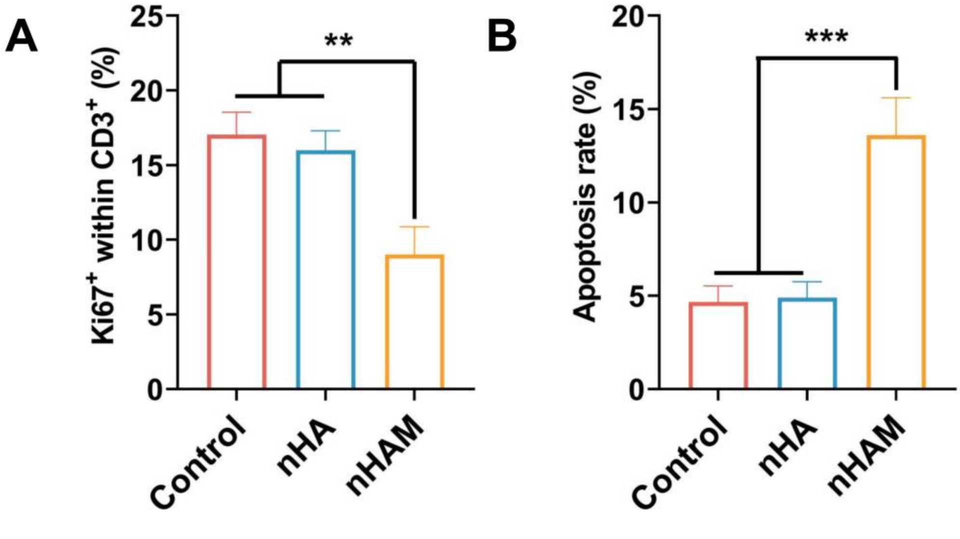

Figure S10. A) the fraction of $CD3^+Ki67^+$ T cells after incubation with nHA, nHAM and blank control (**, p < 0.01). B) the apoptosis rate of $CD3^+$ T cells after incubation with nHA, nHAM and blank control (***, p< 0.001).

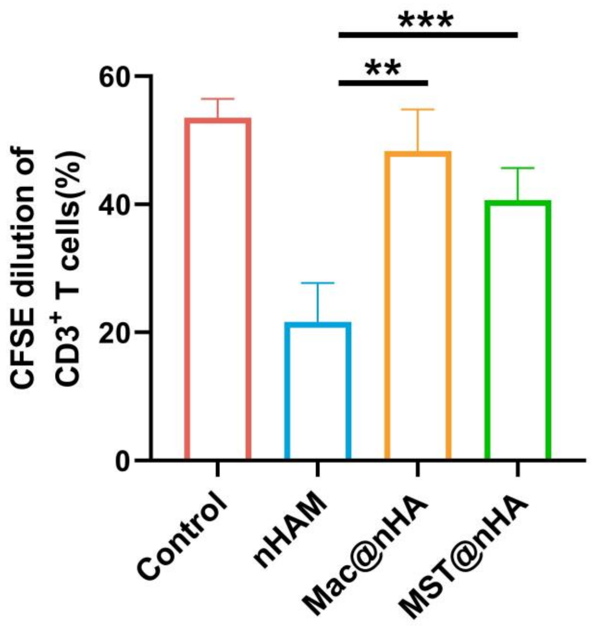

Figure S11. The quantitative analysis of the CFSE dilution shows a significant inhibition for T cell proliferation in different treatment groups (**, $p < 0.01$; ***, $p < 0.001$).

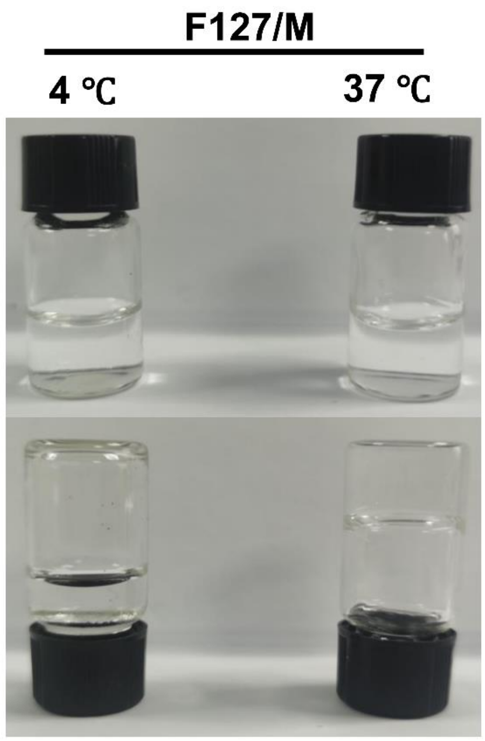

Figure S12. Image of the flow state of F127/M at 4 ℃ and 37 ℃ showing temperature sensitive characteristic.

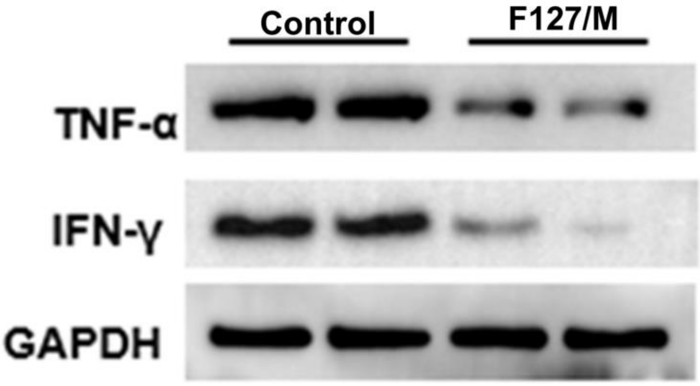

Figure S13. WB detected the protein level of IFN-γ and TNF-α after F127/M treatment.

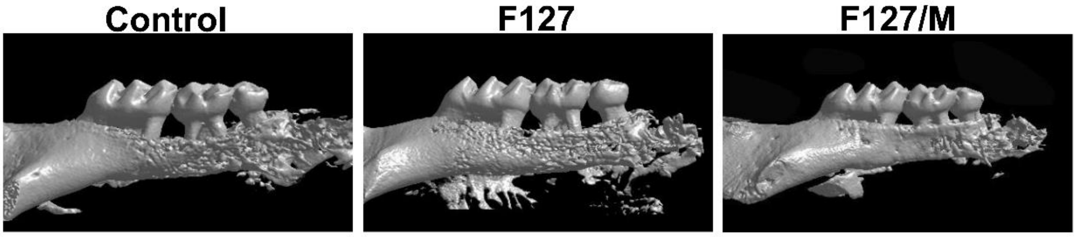

Figure S14. Representative micro-CT graphs of mouse maxillae with F127, F127M or PBS treatment.

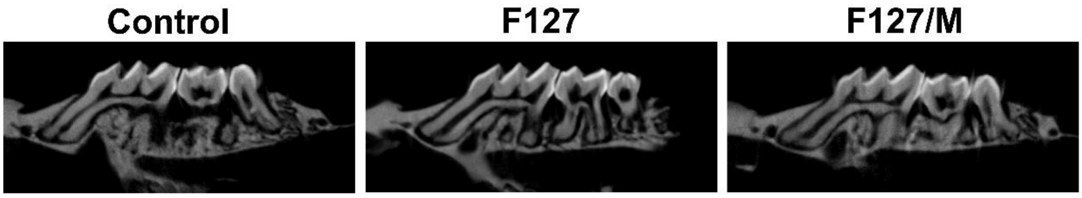

Figure S15. Representative coronal two-dimensional (2D) graphs of mouse maxillae with F127, F127M or PBS treatment.

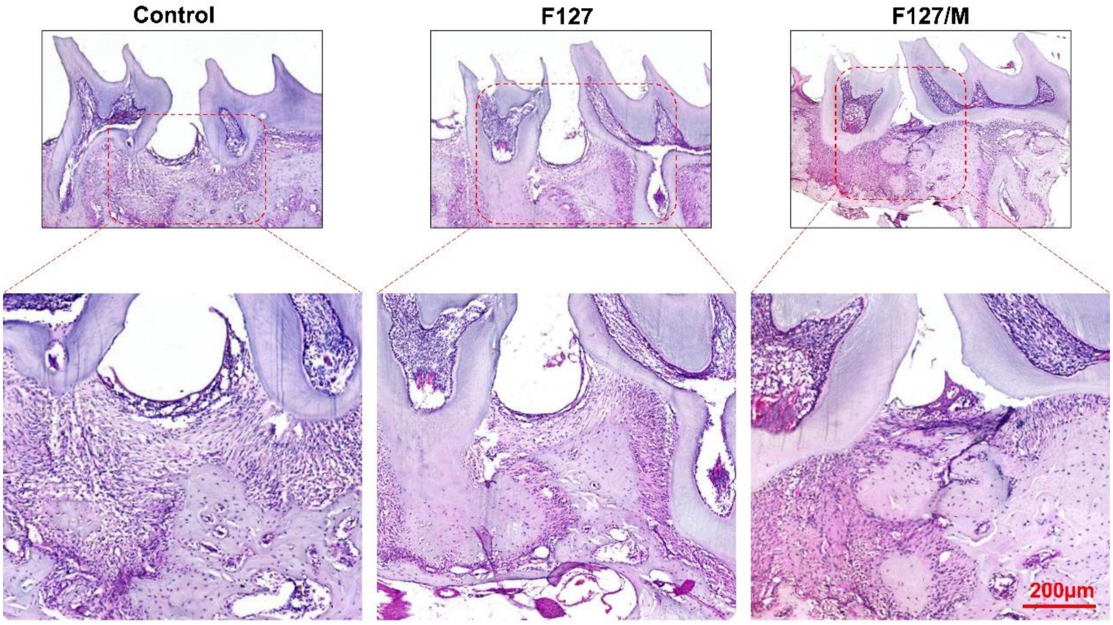

Figure S16. Representative H&E staining of the molar area of maxillae tissue with F127, F127M or PBS treatment.

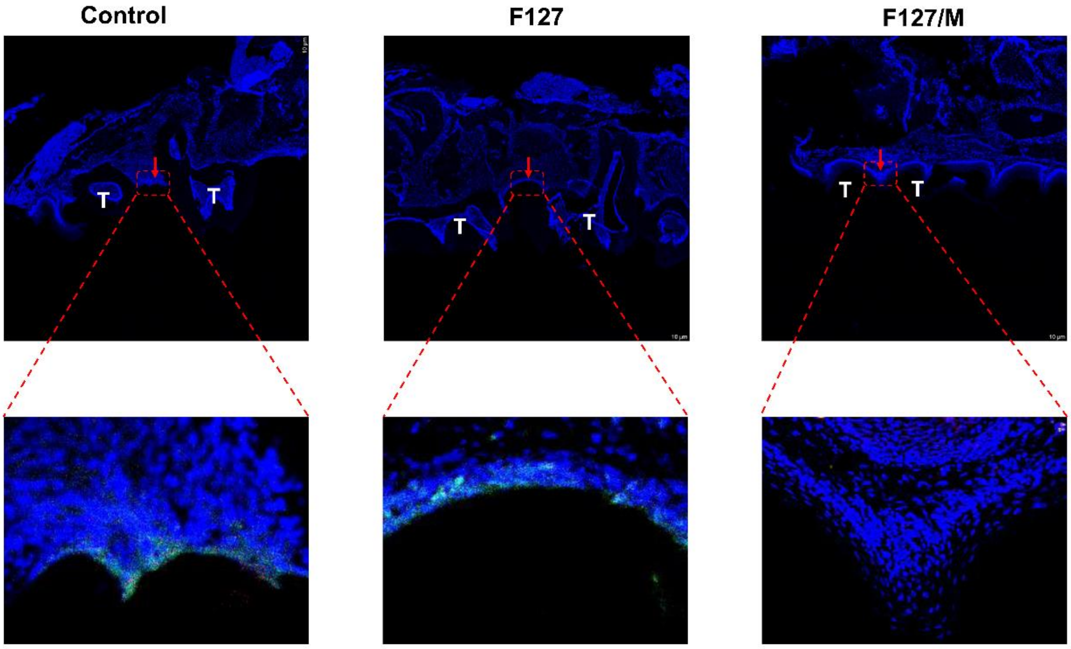

Figure S17. Representative immunofluorescence staining for CD4 (green) and CD8 (red) of the molar area of maxillae tissue with F127, F127M or PBS treatment.

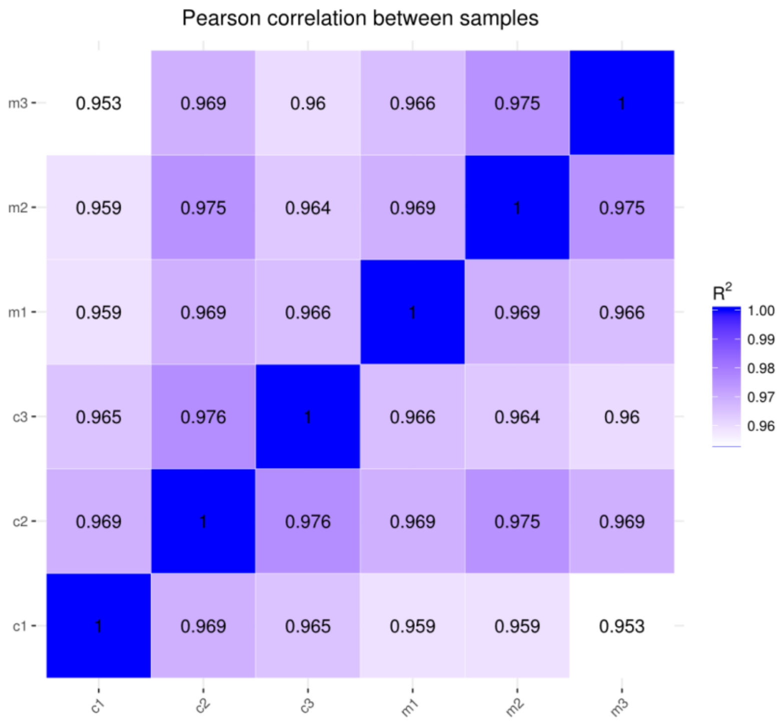

Figure S18. The person correlation between samples.

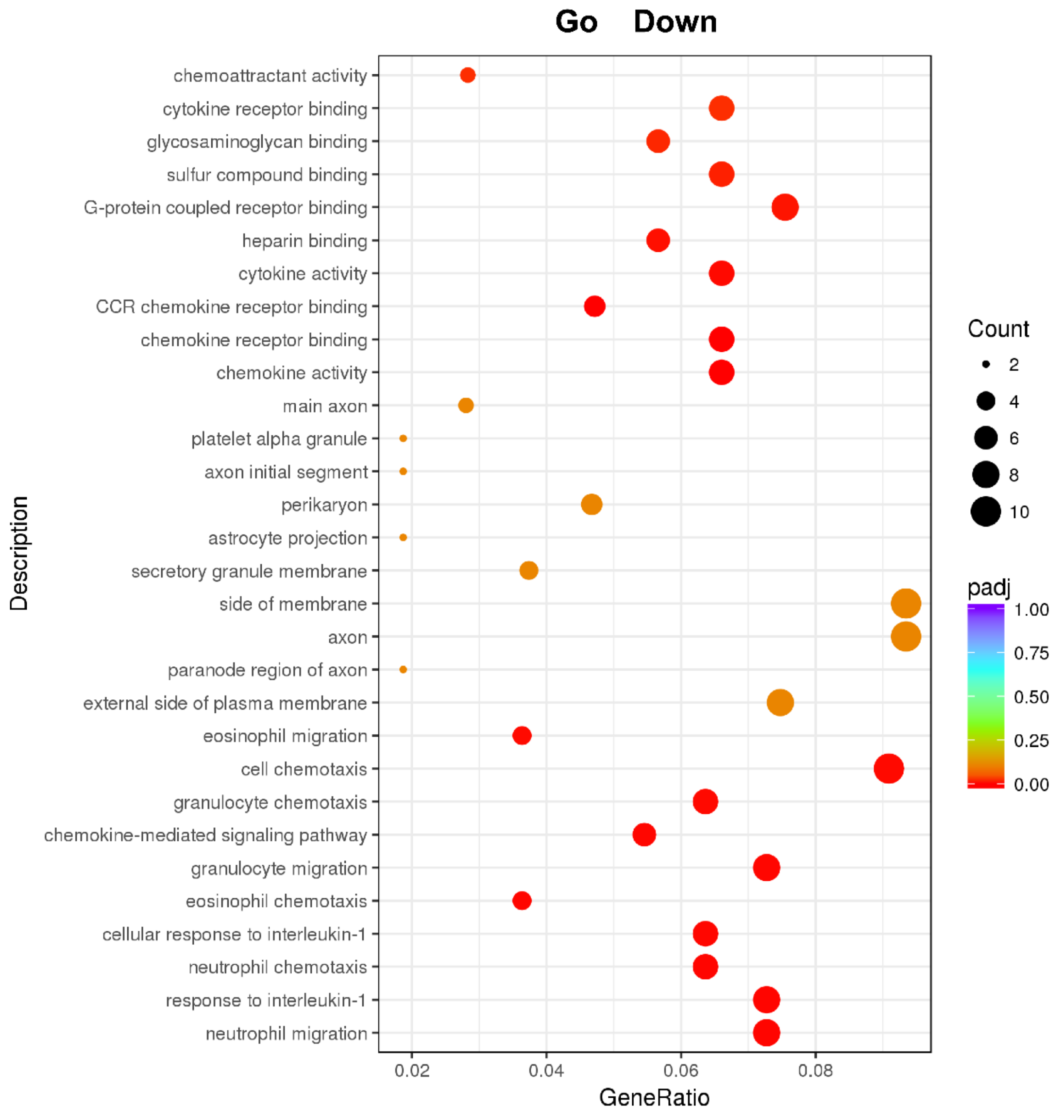

Figure S19. Down-regulative gene ontology enriched in nHAM treatment and control group.

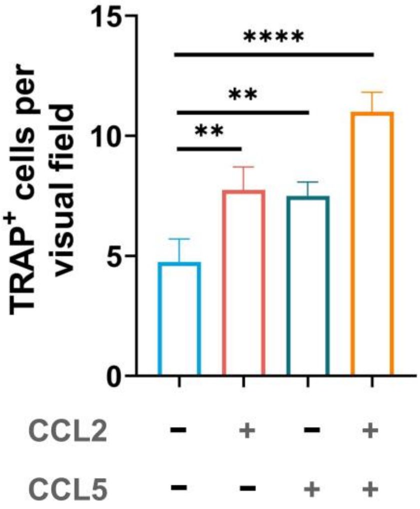

Figure S20. The quantitative bar graph about TRAP$^+$ cell for differentiation of macrophages into osteoclasts in CCL2 or CCL5 treatment group (**, $p < 0.01$; ****, $p < 0.0001$).

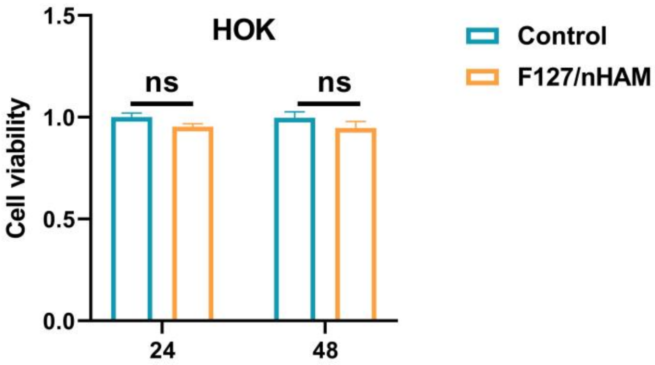

Figure S21. The result of CCK8 in HOK.

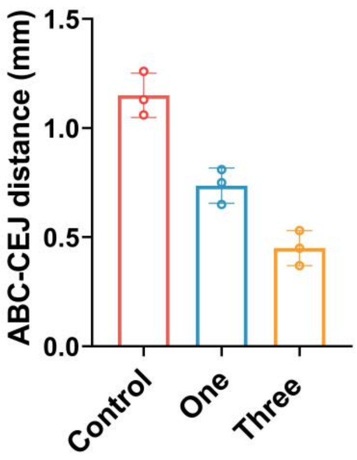

Figure S22. The quantitative analysis of ABC-CEJ distance after three months in F127/nHAM after one and three treatments.

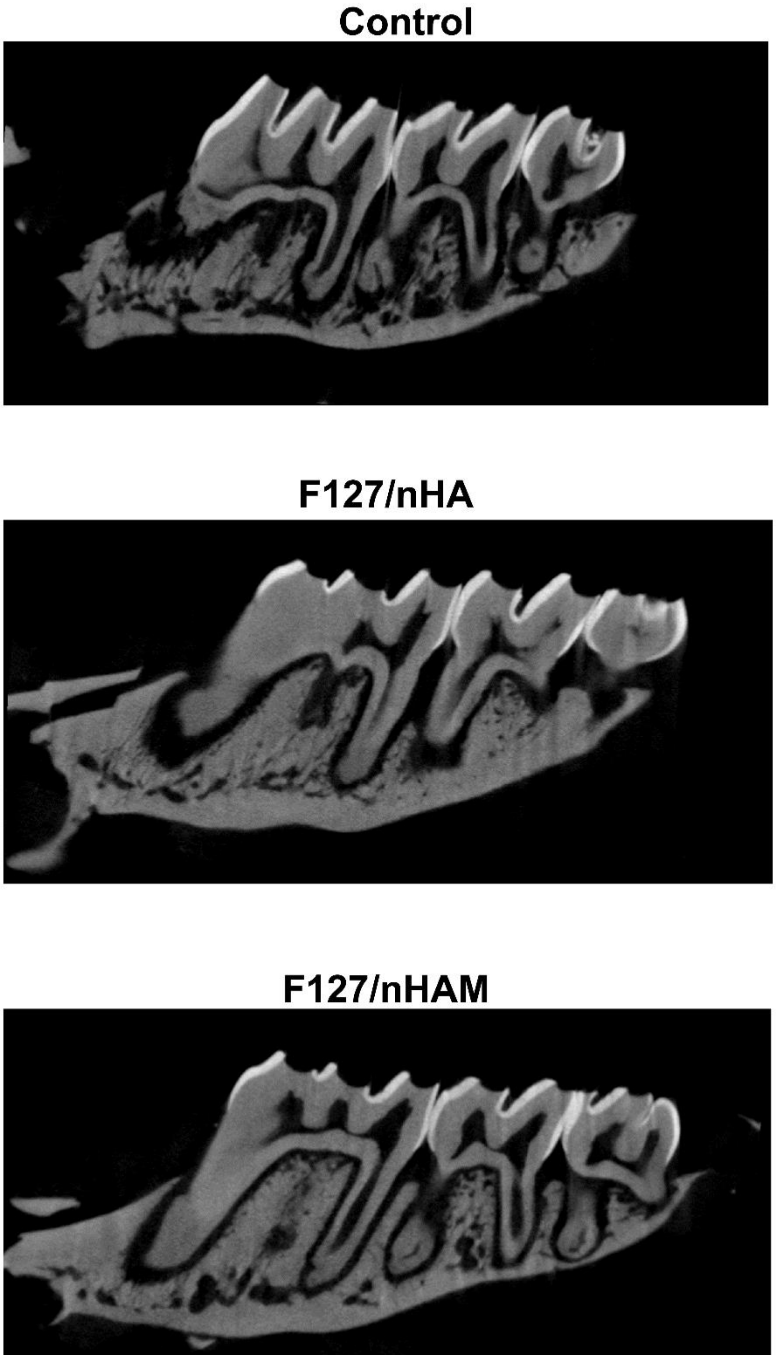

Figure S23. Representative coronal two-dimensional (2D) graphs of rat maxillae with F127/nHA, F127/nHAM or PBS treatment.

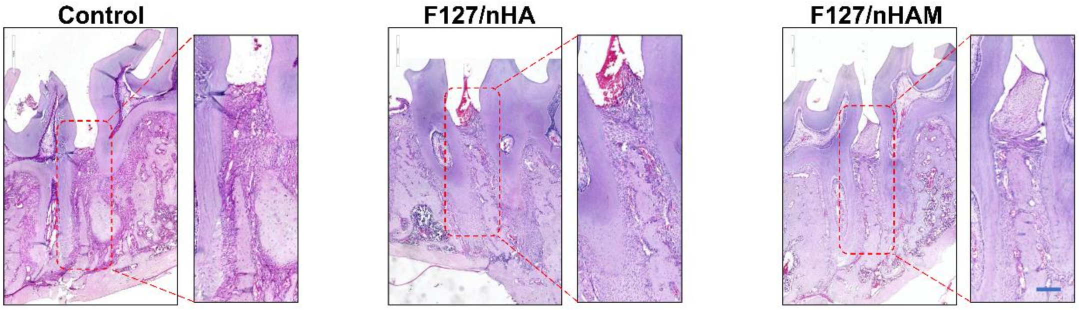

Figure S24. Representative H&E staining of the molar area of maxillae tissue with F127/nHA, F127/nHAM or PBS treatment (scale bar = 400 µm).

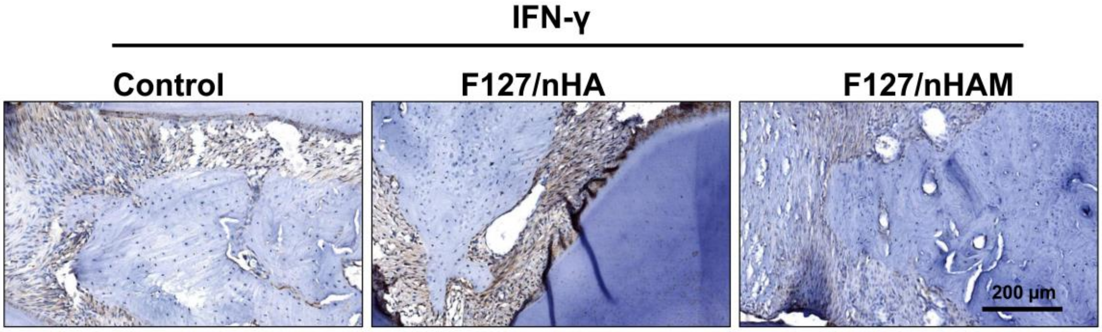

Figure S25. Representative IFN--γ staining of the molar area of maxillae tissue with F127/nHA, F127/nHAM or PBS treatment (scale bar = 200 µm).

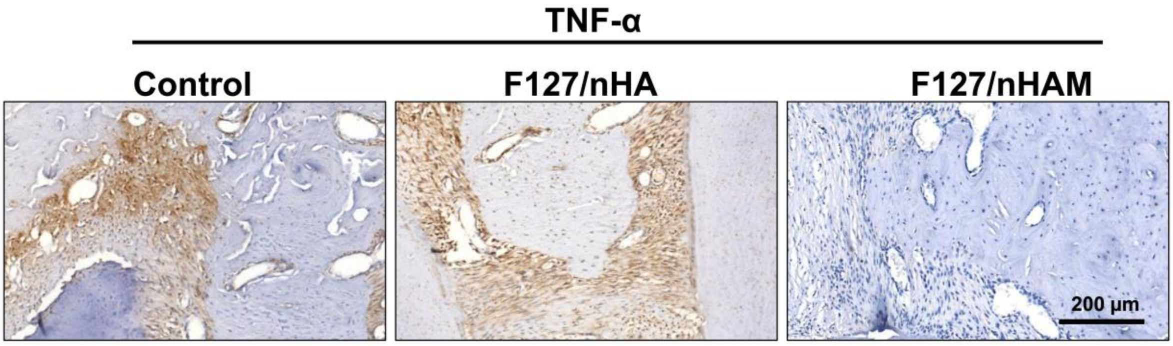

Figure S26. Representative TNF-α staining of the molar area of maxillae tissue with F127/nHA, F127/nHAM or PBS treatment (scale bar = 200 µm).

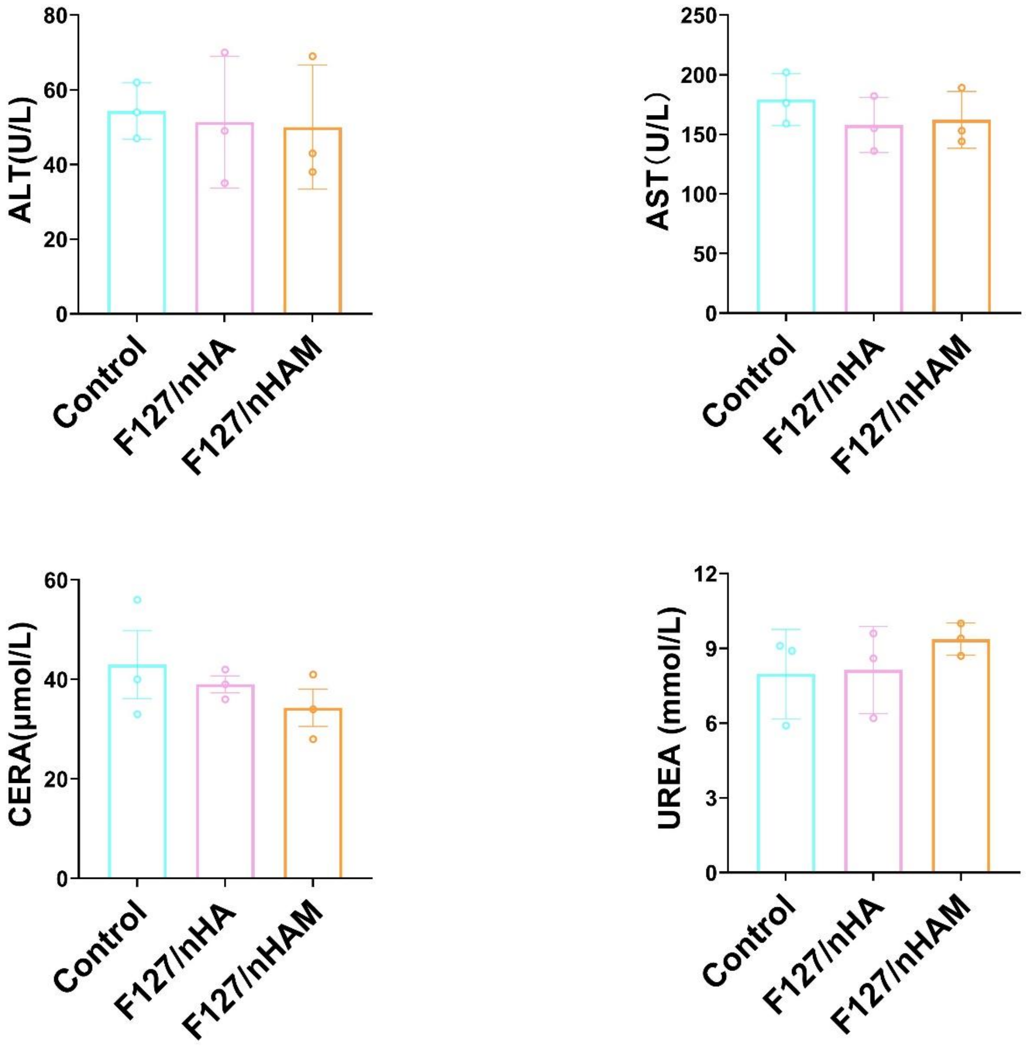

Figure S27. Blood biochemistry analysis (ALT, AST, CERA and UREA) at the end of the experiment with F127/nHA, F127/nHAM or PBS treatment.
